# Human M1 macrophages express unique innate immune response genes after mycobacterial infection to defend against tuberculosis

**DOI:** 10.1038/s42003-022-03387-9

**Published:** 2022-05-19

**Authors:** Arshad Khan, Kangling Zhang, Vipul K. Singh, Abhishek Mishra, Priyanka Kachroo, Tian Bing, Jong Hak Won, Arunmani Mani, Ramesha Papanna, Lovepreet K. Mann, Eder Ledezma-Campos, Genesis Aguillon-Duran, David H. Canaday, Sunil A. David, Blanca I. Restrepo, Nhung Nguyen Viet, Ha Phan, Edward A. Graviss, James M. Musser, Deepak Kaushal, Marie Claire Gauduin, Chinnaswamy Jagannath

**Affiliations:** 1 Department of Pathology and Genomic Medicine, Houston Methodist Research Institute, Weill-Cornell Medicine, Houston, TX USA; 2 Department of Pharmacology and Toxicology, University of Texas Medical Branch, Galveston, TX USA; 3 Department of Obstetrics, Gynecology and Reproductive Sciences, UTHSC, Houston, TX USA; 4 Centro Regional de TB, Secretaría de Salud de Tamaulipas, Reynosa, Mexico; 5 Division of Infectious Disease, Case Western Reserve University Cleveland VA, Cleveland, OH USA; 6 Virovax, LLC, Adjuvant Division, Lawrence, Kansas USA; 7 UT School of Public Health, Brownsville, and STDOI, UT Rio Grande Valley, Brownsville, TX USA; 8 National Lung Hospital, Ha Noi, Vietnam; 9 Center for Promotion of Advancement of Society, Ha Noi, Vietnam; 10 Southwest National Primate Research Center, Texas Biomedical Research Institute, San Antonio, TX USA

**Keywords:** Antigen processing and presentation, Immunology, Bacterial infection

## Abstract

*Mycobacterium tuberculosis* (Mtb) is responsible for approximately 1.5 million deaths each year. Though 10% of patients develop tuberculosis (TB) after infection, 90% of these infections are latent. Further, mice are nearly uniformly susceptible to Mtb but their M1-polarized macrophages (M1-MΦs) can inhibit Mtb in vitro, suggesting that M1-MΦs may be able to regulate anti-TB immunity. We sought to determine whether human MΦ heterogeneity contributes to TB immunity. Here we show that IFN-γ-programmed M1-MΦs degrade Mtb through increased expression of innate immunity regulatory genes (*Inregs*). In contrast, IL-4-programmed M2-polarized MΦs (M2-MΦs) are permissive for Mtb proliferation and exhibit reduced *Inregs* expression. M1-MΦs and M2-MΦs express pro- and anti-inflammatory cytokine-chemokines, respectively, and M1-MΦs show nitric oxide and autophagy-dependent degradation of Mtb, leading to increased antigen presentation to T cells through an *ATG-RAB7*-cathepsin pathway. Despite Mtb infection, M1-MΦs show increased histone acetylation at the *ATG5* promoter and pro-autophagy phenotypes, while increased histone deacetylases lead to decreased autophagy in M2-MΦs. Finally, Mtb-infected neonatal macaques express human *Inregs* in their lymph nodes and macrophages, suggesting that M1 and M2 phenotypes can mediate immunity to TB in both humans and macaques. We conclude that human MФ subsets show unique patterns of gene expression that enable differential control of TB after infection. These genes could serve as targets for diagnosis and immunotherapy of TB.

## Abstract (web summary)

Khan *et al*. use human blood and a macaque model of tuberculosis (TB) to assess whether macrophage heterogeneity contributes to TB immunity. They demonstrate that human macrophages show unique patterns of gene expression that enable differential control of TB after infection.

## Introduction

*Mycobacterium tuberculosis* (Mtb) causes 8 million new cases of tuberculosis (TB) and about 1.5 million human deaths each year^1^. Mtb is a highly infectious organism, but nearly 90% of those infected do not develop active disease, leaving a third of the human population latently infected (LTBI). Though Mtb antigens trigger T cell-mediated responses, emerging studies indicate that innate immunity also plays a role in preventing active TB^2^.

Macrophages (MΦs) are a key element for both innate and adaptive immunity. T cell-derived cytokines like IFN-γ can activate MΦs to kill and degrade Mtb through nitric oxide (NO) and reactive oxygen species (ROS)-dependent mechanisms and phagosome-lysosome fusion^3^. Alveolar MΦs are the first immune cells to encounter Mtb after aerosolized infection and they contribute to the granuloma response in the lung parenchyma during TB^4,5^. Lung granulomas can contain Mtb, but necrotic granulomas can lead to cavities and subsequent dissemination of Mtb^6^. The anti-microbial mechanisms of mouse but not human MΦs are well characterized. Indeed, fewer studies have been reported using human MΦs compared to mouse MΦs. Human MΦs secrete NO at lower levels, show marked functional and phenotypic heterogeneity among tissues, and likely differ in their anti-microbial mechanisms^7–9^. This difference is reflected by the near-uniform susceptibility of most mouse strains to low-dose aerosol infection with Mtb versus the differential susceptibility of humans to active disease.

MΦs can be differentiated into a pro-inflammatory “classical” M1-MΦ phenotype, driven by IFN-γ, or an anti-inflammatory M2-MΦs phenotype, driven by IL-4 (M2-a), IL-1β (M2-b), IL-10 (M2-c), IL-13 (or a combination of these cytokines). Importantly, MΦ phenotypes exist along a spectrum of these polarizations. Interestingly, cytokines, growth factors, metabolites, and Mtb infection all seem to drive MΦ phenotype in mice^10–14^. During M1-MΦ polarization, mouse MΦs exhibit increased microbicidal capacity, NO production, secretion of pro-inflammatory cytokines, antimicrobial peptide production, phagocytosis, and phagosome-lysosome fusion^15,16^. M1-MΦs are likely to mount an effective response against intracellular pathogens, while M2-MΦs exhibit relatively decreased anti-microbial functions but may aid in tissue repair^8,17,18^. Accordingly, mouse M1- and M2-MΦs show differential susceptibilities to Mtb, broadly characterized by inhibitive M1-MΦs phenotypes and permissive M2-MΦs phenotypes. Although non-human primates (NHP) like rhesus monkeys also show a dose-dependent susceptibility to active TB, their macrophage heterogeneity is yet to be fully characterized.

We hypothesized that a subset of human MФs can acquire enhanced anti-TB function upon cytokine exposure and that this exposure partially explains the development of LTBI. Human alveolar MΦs (AMΦs) also express M1- and M2- phenotypes^19^ and, although AMΦs are the first to encounter Mtb after aerosol infection^5^, alveoli are also rapidly infiltrated by interstitial MФs (IMΦs), and peripheral blood mononuclear cells (PBMCs) including T cells, neutrophils, and dendritic cells (DCs) from the vasculature following infection. Thus, IMΦs derived from PBMCs likely differentiate locally into M1- and M2- phenotypes following exposure to cytokine milieus upon arrival.

Herein, we demonstrate that human M1-MΦs upregulate multiple innate immunity regulatory genes and gene clusters (*Inregs*), including *ATGs, RAB* GTPases, and cathepsin proteolytic enzymes to degrade Mtb more effectively than M2-MΦs. Further, compared to M2-MΦs, M1-MΦs processed and presented Mtb antigen to CD4 T cells more efficiently ex vivo. We also found that Mtb epigenetically alters histones associated with *ATG5*, which decreased autophagy in M2-MΦs. In contrast, M1-MΦs retained their anti-mycobacterial autophagy by selectively expressing histone deacetylases (HDACs). Finally, we demonstrate that Mtb-infected infant macaque-derived lymph nodes and MΦs show transcriptional responses like those observed in human MΦs. We propose that cytokine-exposed human MΦs can differentiate into M1- and M2- subsets during natural infection and that the differential expression of *Inregs* regulates the functional heterogeneity of human and NHP MΦs, explaining in part the differential susceptibility of humans and NHPs to TB.

## Results

### Human peripheral blood and cord blood-derived MФs polarized to M1- (IFN-γ) and M2- (IL-4) phenotypes differentially regulate the growth of intracellular Mtb through nitric oxide and autophagy

To characterize human MФ heterogeneity, we used IFN-γ or IL-4 to polarize PBMC-derived and GM-CSF cultured MФs into M1- and M2-MФs phenotypes, respectively^20,21^, and confirmed surface expression of CD80(M1)/CD206(M2) using flow cytometry. We further confirmed the ability of the cells to phagocytose Mtb with a colony-forming unit (CFU) assay^22,23^. We activated cells for 5 days using a low-dose cytokine activation protocol, which allowed the cells to retain >90% viability through day 7 post Mtb infection. Though Mtb-infected M1-MФs survived through day 21 post Mtb infection, we observed a progressive loss of viability. M2- and M0-MФs showed marked viability loss 10 days post-infection. The lack of apoptosis in Mtb-infected or naïve cultures was confirmed using a Tunel assay between days 1–7.

**Fig. 1:**
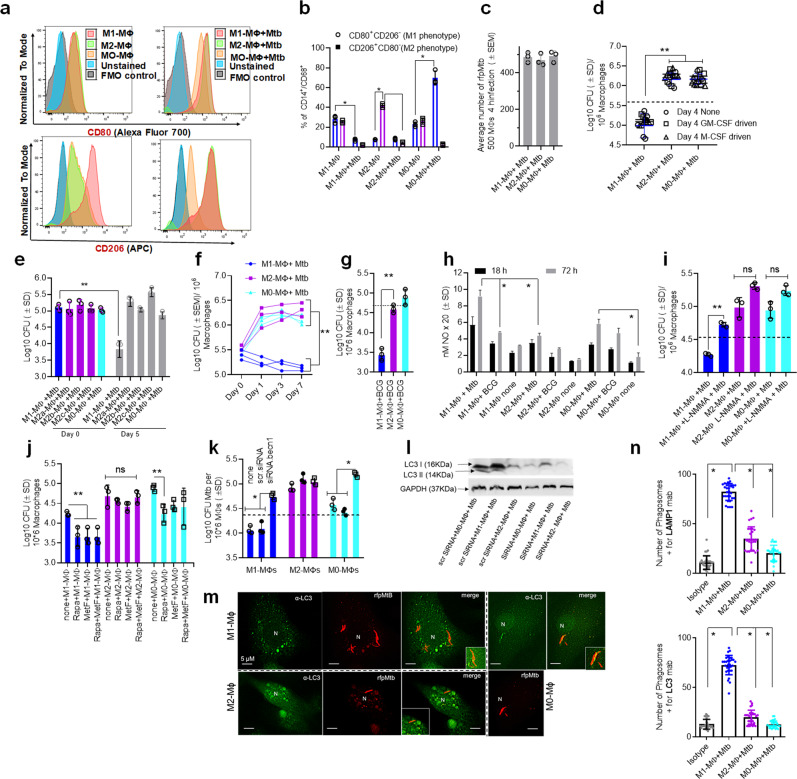
Human umbilical cord and peripheral blood-derived macrophages show heterogeneity in mycobacterial killing associated with oxidants and autophagy. Human cord blood (CBM) or healthy donor PBMC-derived MФs were cultured in the presence of either recombinant human IFN-γ (M1; 10 ng/mL) or human IL-4 (M2; 10 ng/mL) for 5 days and rested for 2 days. Untreated cells were M0-MФs. **a**, **b** Surface expression of receptors by CBM-derived naïve and *Mycobacterium tuberculosis* (Mtb; H37Rv)-infected M1- (CD80^+^/CD206^−^) and M2-MФs (CD80^−^/CD206^+^) on day 3 using flow cytometry and quantitation (*p < 0.01 *t* test); gating strategy shown Supplemental Fig. 1. **c** CBM-derived differentiated MФs were infected with Mtb for 4 h followed by microscopic counts of *rfp*-labeled MtbH37Rv to determine uptake verified using CFU counts. **d** PBMC-derived MФs from five healthy donors were differentiated using the indicated cytokines followed by infection with Mtb and CFU assay on day 4. Each point represents one donor (**p < 0.05; Kruskal–Wallis test). **e** PBMC-derived MФs were differentiated using GM-CSF (M1), IL-4 (M2a), IL-1β M2b), IL-10 (M2c) or left intreated (M0) followed by Mtb infection and CFU assay on day 4 (**p < 0.007). **f** CBM-derived, cytokine differentiated MФs were infected with Mtb followed by CFU assay over time (**p < 0.006; data from 3 experiments shown). **g** CBM-derived, differentiated MФs were infected with *M. bovis* BCG followed by CFU assay on day 4 (**p < 0.005). **h** CBM-derived and differentiated uninfected MФs or those infected with Mtb or BCG were incubated and at indicated time points, cultures were tested for nitrite using diaminofluorescein diacetate and fluorometry (*,**p < 0.005, *t* test). QPCR for mRNA of iNOS and protein are shown in Supplemental Fig. 3a and reactive oxygen species levels in Supplementary Fig. 3b. **i** MФs infected with Mtb as in panel h were incubated in NMMA (0.5 mM; N-monomethyl l-arginine) followed by CFU assay on day 3 (**p < 0.009). **j** CBM-derived, differentiated MФs infected with Mtb were incubated with 10 µM Rapamycin, 100 µM Metformin or their combination followed by CFU assay on day 3 (**p < 0.009). **k** CBM-derived, M1-, M2- and M0-MΦs were treated in the presence or absence of siRNA vs. beclin1 (*ATG6*) or its scrambled control followed by infection with Mtb and CFU counts on day 3 (*p < 0.007). Blot validation of Knockout using siRNA vs. beclin1 (*ATG6*) is shown in Fig. 3h. **l** MΦ lysates of panel *k* collected at 18 h were analyzed using western blots for the lipidation of microtubule-associated light chain 3 (LC3). Lipidation is indicated by LC3-II. **m** CBM-derived MΦs were infected with *rfp*MtbH37Rv and stained for an LC3 autophagy marker or LAMP1 lysosome marker using specific antibodies, Alex-Fluor485 conjugates, and imaged using confocal microscopy. Panels illustrate LC3 colocalization; LAMP1 stains using *gfp*MtbH37Rv is shown in Supplementary Fig. 3c. **n** Quantification of phagosomes colocalizing with LC3 are shown using an N90 Nikon fluorescence microscope (IF) and Metaview software (*p < 0.004, *t* test). For panels (**b**–**c**–**e**–**f**–**g**–**h**–**i**–**b**–**k**–**n**), *p*-values were calculated using a one-way ANOVA with Tukey’s post-hoc test; one of 2–3 similar experiments shown. CFU or IL-2 assays had triplicate wells plated per group or donor. Panels (**d**, **g**, **i**–**k**) horizontal dotted lines indicate day 0 CFU (4 h post-infection CFU). All Mtb CFU experiments used MOI of 1.

M1-MФs were CD80^+^/CD206^−^ and M2-MФs were CD80^−^/CD206^+^ (Fig. 1a, b) (gating strategy shown in Supplementary Fig. 1). Both phenotypes showed comparable phagocytic uptake of Mtb; Fig. 1c shows *rfp*Mtb uptake immediately after a 4 h (hour) infection. Consistent with previous observations, M1-MФs secreted many pro-inflammatory cytokines (IL-12, TNF-α, IL1-β, IL-8, GM-CSF, IL1-α, IL-17) and chemokines (CCL2, CCL13, CCL18, CCL22), while M2-MФs secreted anti-inflammatory cytokines (IL-4, IL-6, IL-10) (Supplementary Fig. 2). We cultured PBMCs from five healthy donors using either GM-CSF or M-CSF followed by M1- and M2- differentiation and an Mtb growth assay. GM-CSF and M-CSF-cultured MФs showed similar Mtb growth profiles following polarization (Fig. 1d). Unlike previous studies, we did not use lipopolysaccharide (LPS) in combination with IFN-γ to activate M1-MФs as LPS is a Gram-negative bacterial ligand inducing pleiotropic effects^23^, and Mtb can itself activate TLR-4^24^. We also found that LPS can trigger autophagy through TLR-4^25^. Although subsets of M2-MФs are known^26^, we did not observe significant differences in Mtb infection assay profiles of Mtb-infected subsets (Fig. 1e).

Monocytes from cord blood and adult PBMCs are similar. To obtain a consistently high yield of MФs required to elucidate the molecular mechanisms underlying MФ heterogeneity, we optimized an ex vivo culture system using cord blood-derived monocytes (CBMs) differentiated into M1- and M2-MФ phenotypes. CBMs cultured in medium with GM-CSF were rested (M0-MФ) or differentiated into either M1- or M2-MФs using IFN-γ and IL-4, respectively^21^. CBM-derived M1- and M2-MФs restricted Mtb growth (Fig. 1f) to an extent similar to that observed in PBMC-derived M1- and M2-MФs (Fig. 1d, e). M1-MФs also significantly restricted Bacillus Calmette–Guérin (BCG) growth compared to M2-MФs (Fig. 1g**)**. Although a few studies have shown that human M1-MФs kill Mtb better than M2-MФs, we emphasize that M1-polarization was achieved using IFN-γ and LPS^20^; thus, LPS-induced pleiotropic effects cannot be ruled out.

We performed a fluorometric nitrite assay on Mtb-infected MФs^27^ and found that Mtb- or BCG-infected M1-MФs produced higher levels of nitrite compared to M2- or M0-MФs at 72 h post-infection (Fig. 1h). In a separate experiment, we blocked NO synthesis by incubating Mtb-infected MФs with N-monomethyl l-arginine and assessed Mtb growth. N-monomethyl L-arginine blockade of NO enhanced Mtb growth in M1- but not M2- or M0-MФs (Fig. 1i). Interestingly, Mtb infected M1-MФs expressed higher levels of iNOS mRNA compared to Mtb-infected M2-MФs (Supplementary Fig. 3a). Of interest, iNOS protein was detectable in Mtb-infected but not uninfected MФs, although no quantitative differences were found in protein levels after Mtb infection between M1-, M2- or M0-MФs. Even though uninfected MФs were negative for iNOS protein using blots, fluorometry was much more sensitive and detected basal levels of NO. Similar to NO, Mtb and BCG-infected M1-MФs produced higher levels of whole cell ROS detected using DCFDA compared to Mtb-infected M2- or M0-MФs^28^; uninfected M1-MФs also showed elevated mitochondrial ROS detected using MitoROS (Supplementary Fig. 3b). It remains unclear whether *gp91-phox* and phagosome-dependent generation of ROS occurred in MФs and needs investigation.

To determine if autophagy contributes to Mtb degradation, we performed dose titrations using Rapamycin and Metformin, two drugs that induce degradation of Mtb in naïve MФs. At a dose of 10 µM, Rapamycin reduced the Mtb colony counts (CFUs) in M1-, M2 and M0-MФs (≥0.5 log_10_ decrease in Mtb counts over 3 days; *p* < 0.01 *t* test), while 100 µM Metformin was effective only in M1-MФs. A suboptimal dose of 1 µM Rapamycin and 100 µM Metformin increased bactericidal function in M1- and M0- but not M2-MФs, although the drugs did not synergize (experiment performed in duplicate) (Fig. 1j). Because Rapamycin is a known inducer of autophagy, we next treated Mtb-infected M1-, M2- and M0-MФs with siRNA vs. beclin1 (*ATG6*) (autophagy blockade) and assessed changes in Mtb proliferation using a CFU assay. siRNA vs. beclin1 enhanced Mtb growth in M1-and M0-MФs but not M2-MФs (Fig. 1k).

As autophagic flux is indicated by the localization of LC3 on autophagolysosomes (APLs) containing Mtb, which also label for LAMP1 and Rab7^29^, we next assessed LC3 lipidation. Increased LC3 lipidation was observed in M1- and M0-MФs (Fig. 1l), although only the *rfp*Mtb phagosomes of M1-MФs demonstrated increased colocalization with both LC3 and LAMP1 (Fig. 1m, n). Despite LC3 lipidation (Fig. 1m, n), *rfp*Mtb phagosomes of M0-MФs were less enriched for LAMP1 (Fig. 1m) (Supplementary Fig. 3c). suggesting a lack of fusion with APLs, a finding consistent with reports that Mtb phagosomes can transiently label with LC3^30^. Together, these data strongly suggest that human M1- and M2-MФs are heterogeneous with differing NO and ROS levels, and autophagic sorting of mycobacteria. These differences explain the Mtb-inhibitive versus Mtb-permissive states of M1-MФs and M2-MФs, respectively.

### Mtb infection induces differential expression of innate immunity regulating genes (Inregs) in human M1- and M2-MФs

**Fig. 2:**
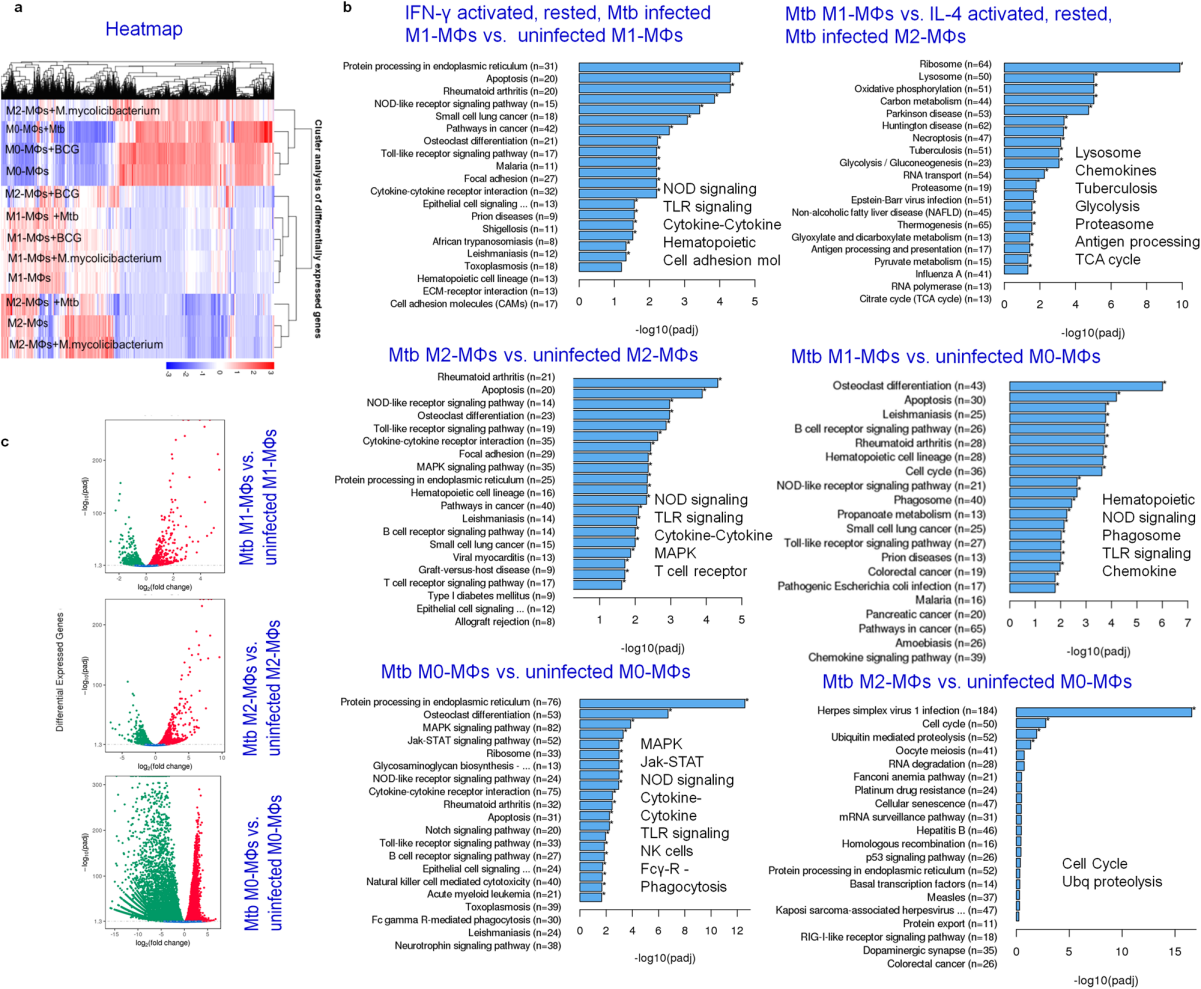
*Mycobacterium tuberculosis* (Mtb) induces differential expression of innate immunity regulating genes (*Inregs*) in human M1- and M2-MФs. CBM-derived uninfected M0-, M1- and M2-MΦs or those infected separately with Mtb H37Rv, BCG or *Mycolicibacterium smegmatis* were analyzed using RNAseq (*Novogene Inc*., USA) at 18 h (MOI of 1; *n* = 2 samples per group; analysis done twice). **a** Heat maps highlight Mtb-induced differential gene expression. BCG and *M. smegmatis* profiles are included but not highlighted. **b** Kyoto Encyclopedia of Genes and Genomes profiles of Mtb-infected M1-, M2- and M0-MΦs vs. uninfected MΦs show differential enrichment of *Inregs* and pathways in the context of TB control (*p-values < 0.00001; *Clusterprofiler* workflow). Only gene clusters showing a significant *p*-value (<0.00001) are highlighted. **c** Volcano plots indicate the Mtb-induced differential gene expression. Additional gene expression profiles and *Clusterprofiler* pathway analysis are shown in Supplementary Figs. 4–7. RNAseq and pathway analysis by *Novogene* is illustrated in Supplementary Fig. 13. All Mtb CFU experiments used MOI of 1.

To better understand the molecular basis of phenotypic and functional heterogeneity in MФs, we subjected naïve and Mtb-infected M1-, M2-, and M0-MФs to RNAseq analysis. We also separately infected MФs with avirulent BCG vaccine and nonpathogenic *Mycolicibacterium smegmatis* and analyzed cells in parallel for gene expression (heat maps include BCG and *M. smegmatis*) (Fig. 2a). Heat maps indicate that M1-, M2-, and M0-MФs responded differently to Mtb infection, with a marked difference between M1- and M2-MФs responses. Gene clusters that control anti-mycobacterial immune responses including, NOD/TLR-dependent signaling mechanisms, cytokine-chemokines, FcG-receptor-mediated signaling, and genes regulating the sorting of the pathogen from phagosomes to lysosomes were differentially expressed and are highlighted in Fig. 2b, c. Compared to M2-MФs, M1-MФs showed an upregulation of genes and pathways that regulate antigen processing (phagosome and/or lysosome, proteasome, antigen processing), hereafter referred to as the *‘*antigen processing transcriptome’ (Fig. 2b). Furthermore, consistent with previous observations that the glycolytic pathway regulates M1-phenotype^31^, glycolysis/gluconeogenesis and tricarboxylic acid cycle cycle pathways were upregulated in Mtb-infected M1-MФs (Fig. 2b). Genes regulating hematopoietic lineage were upregulated only in Mtb-infected M1-MФs. Individual genes that were differentially expressed by Mtb-infected MФs in comparison with their naïve counterparts, including downregulated genes, are further illustrated in Supplementary Fig. 4. Downregulated genes also contribute immunologically to the pathogenesis of TB; for example, ER protein processing was downregulated in M1- vs. M2-MФs (Supplementary Fig. 5). Representative pathway analysis derived from the *Clusterprofiler* workflow is illustrated in the context of up- (red) and down-(green) regulated genes affecting anti-mycobacterial immune responses (Supplementary Figs. 6, 7). Both Mtb-infected M1- and M2-MФs activated multiple genes of the NOD signaling pathway (Supplementary Fig. 6), but only Mtb-infected M1-MФs activated the caspase cascade (*CASP1*), which leads to IL-1β secretion (Supplementary Fig. 2). Likewise, Mtb-infected M1-MФs upregulated multiple genes participating in anti-TB immunity compared to M2-MФs (Supplementary Fig. 7). These data are strikingly different from the transcriptome responses of mouse-derived M1-and M2 MФs and confirm that human MФs respond to Mtb differently than those from mice^32^.

### Mtb-infected human M1- and M2-MФs show differential upregulation of ATGs and accessory genes that regulate autophagy-dependent mycobacterial degradation and antigen presentation

**Fig. 3:**
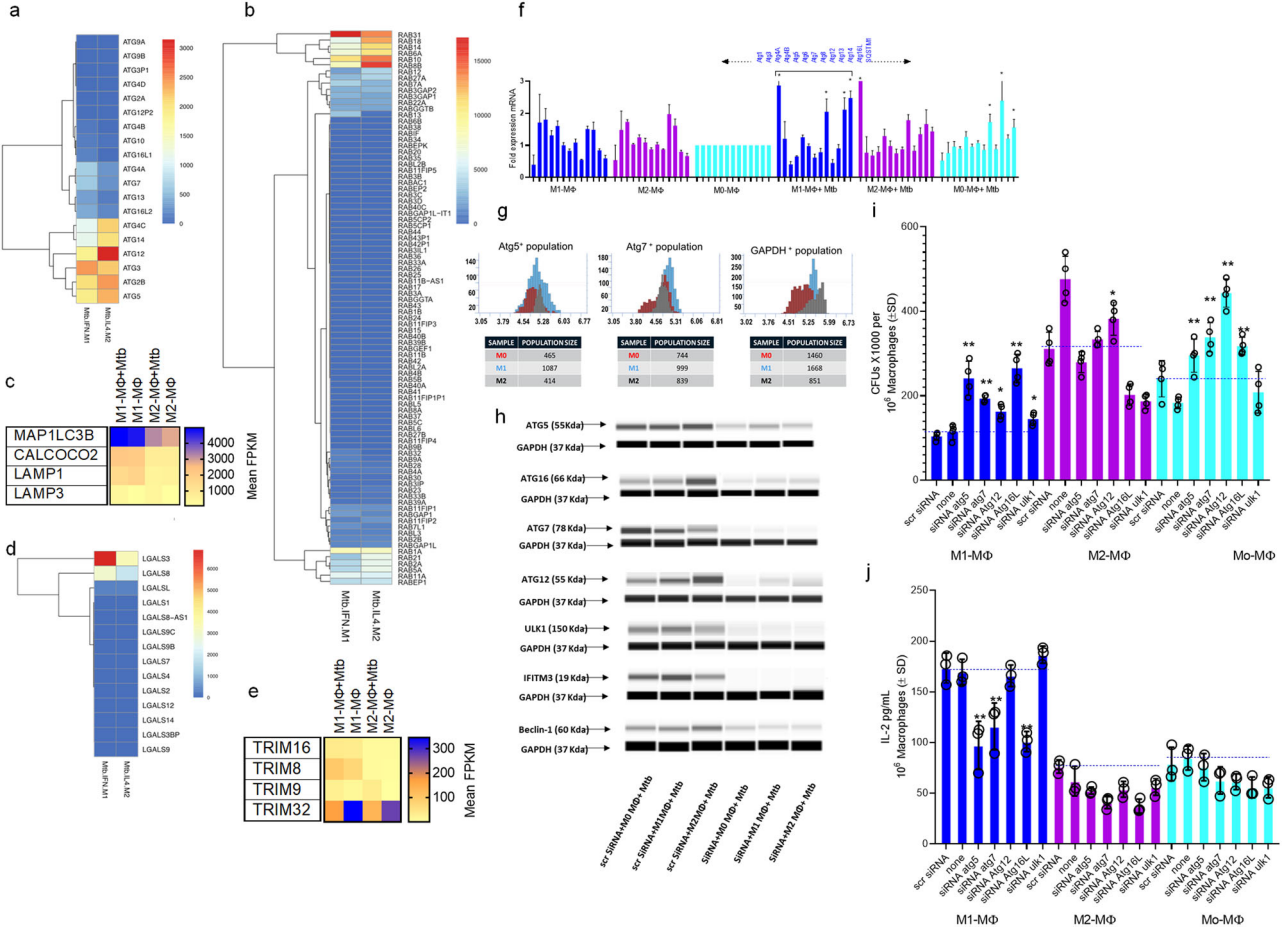
Mtb-infected human M1-, M2-, and M0-MΦs show differential expression of autophagy-regulating *ATGs, RAB GTPases*, Galectins (*LGALS*) and Tripartite-containing motif protein (*TRIM*) encoding accessory genes. **a**–**e** CBM-derived M1-, M2-, and M0-MΦs were subjected to RNAseq before and after Mtb infection as in Fig. 2 at 18 h post Mtb infection. Data are shown for M1- vs. M2-MΦs and transcripts are shown as FPKMs (fragments per kilobase per million reads) for gene clusters (*n* = 2). **f** Quantitative PCR analysis for autophagy-regulating genes (*ATGs*) at 18 h (Mtb-infected vs. naive; *p < 0.01, *t* test, *n* = 2; one of 2 similar experiments). **g** Single cells of uninfected M1-, M2-, and M0-MΦs were dispensed into the wells of the MILO single-cell protein profiler. ATG5 and ATG7 proteins with a GAPDH control were detected using specific antibodies and in situ western blot; quantitation of the number of MΦs expressing proteins are shown. **h** Mtb-infected or naïve M1-, M2-, and M0-MΦs were analyzed for ATG proteins before and after siRNA knockdown including beclin1 (*ATG6*) (Origene, USA) (one of 2 similar experiments shown). Densitometry is shown in Supplementary Fig. 8. **i** Indicated *ATGs* were knocked down using specific duplexes of siRNA (Origene, USA), followed by infection with Mtb and CFU counts on day 3 maintaining >90% viability of MΦs. Baseline CFUs of scrambled siRNA controls are shown by a dotted line (one of 3 experiments shown). **j** siRNA or scrambled siRNA treated replicates of MΦs from panel **i** were overlaid with an Ag85B-derived epitope-specific F9A6-CD4 T cell line for antigen presentation. IL-2 was measured in supernatants using sandwich enzyme-linked immunoassay at 18 h (one of 2 independent experiments shown). Baseline antigen presentation by scrambled siRNA controls is shown by a dotted line. Panels (**i**, **j**): **p < 0.007; *p < 0.009 one-way ANOVA with Tukey’s test. All Mtb CFU experiments used MOI of 1.

Our transcriptome studies indicated that genes regulating antigen processing pathways are upregulated in M1-MФs (Fig. 2; Supplementary Figs. 9, 10). Because knockdown of beclin1 (*ATG6*) a key imitator of autophagy enhanced Mtb proliferation in M1-MФs but not M2-MФs (Fig. 1k), we sought to further characterize the expression of *ATGs* by analyzing their transcripts and proteins before and after Mtb infection. Figure 3a–e shows the differential expression profiles for *ATGs, RAB* GTPases, galectins (*LGALS*), and *TRIMs* in addition to *MAP1LC3B, CALCOCO2, LAMP1*, and *LAMP3*, which encode key proteins mediating APL fusion in Mtb-infected M1- vs. M2-MФs. Mtb-infected M1-MФs expressed more key *ATG*s, including *ATG7*, and *RAB7A*, *MAP1LC3*, *CALCOCO2*, and *LAMP-1* than M2-MФs. Likewise, M1-MФs expressed markedly higher levels of galectins *LGALS*3/8 and *TRIM* 8, *32*, and *38*^33–35^. Figure 3f shows the QPCR profile of *ATG*s.

**Fig. 4:**
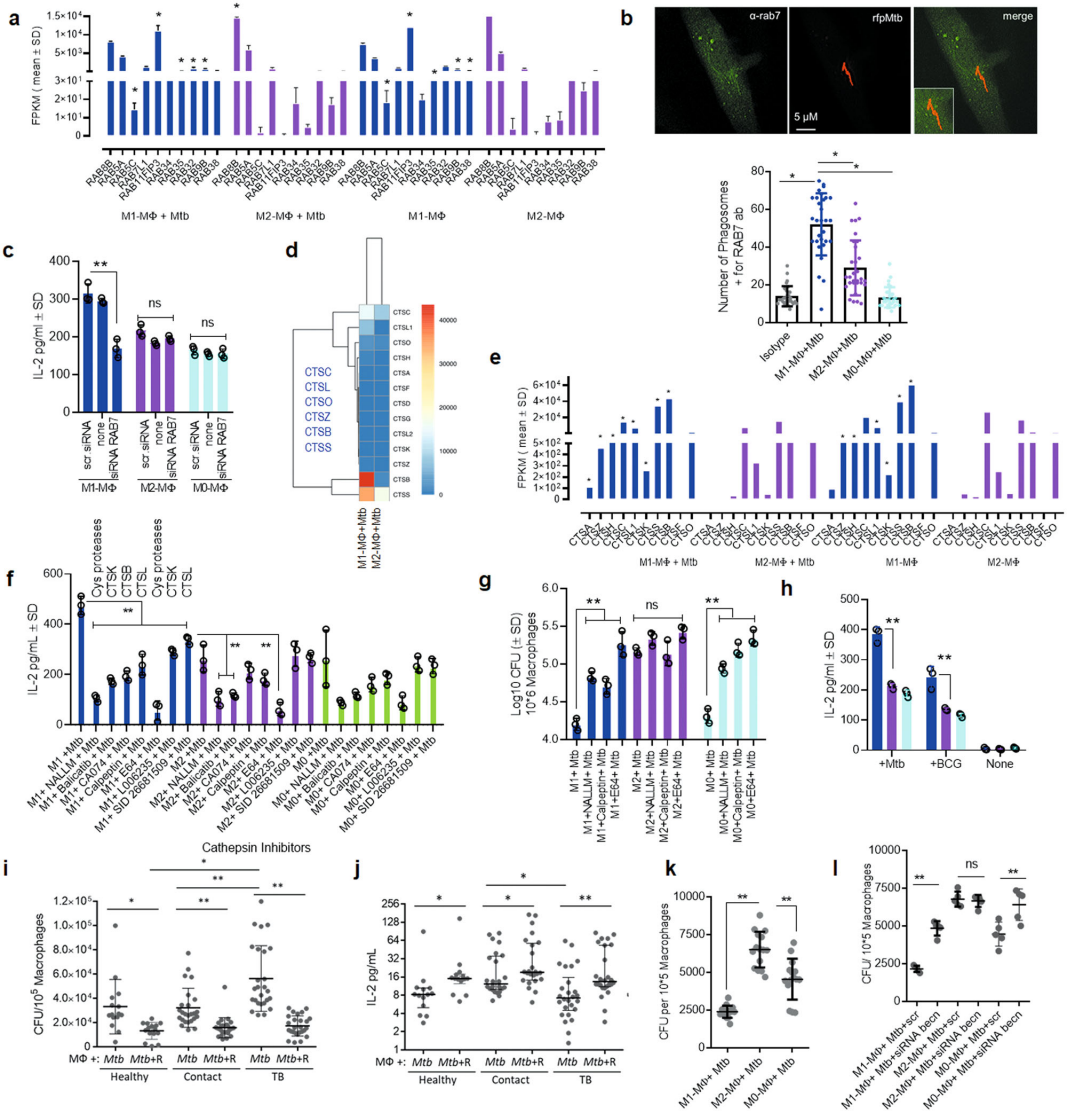
Mtb infection induces differential expression of *RAB GTPases* and cathepsin proteases in human macrophages affecting autophagy-dependent ex vivo antigen presentation to CD4 T cells. **a** Differential expression of *RAB GTPases* and transcripts are shown as FPKMs (fragments per kilobase per million reads) for duplicate samples; *p < 0.01; *t* test. **b** MΦs were infected with *rfp*Mtb, followed by staining with an isotype or specific antibodies to RAB7 or LAMP1 proteins and counterstained using fluorescein isothiocyanate anti-IgG conjugates. Confocal microscopy analysis of phagosomes colocalizing with RAB7 is illustrated (full panels shown in Supplementary Fig. 3c); bar graph indicates quantitation of RAB7 colocalization (*p < 0.009; *t* test; one of 2 similar experiments shown). **c** MΦs indicated were treated with siRNA for *RAB7* or its scrambled control followed by Mtb infection and overlay with F9A6-CD4 T cell line for antigen presentation and IL-2 assay (**p < 0.009; *t* test; one of 2 similar experiments shown). **d**, **e** Differential gene expression of cathepsins (CTS) expressed as FPKMs shown for duplicate samples (*p < 0.01; *t* test). **f** MΦs indicated were treated with non-cytotoxic doses of either CTS specific inhibitors or pan-specific inhibitors followed by Mtb infection and antigen presentation using F9A6-CD4 T cells (**p < 0.009, *t* test; one of 2 similar experiments shown). **g** MΦs treated with CTS inhibitors were infected with Mtb followed by CFU assay on day 5, maintaining >90% viability of MΦs (**p < 0.009, one-way ANOVA with Tukey’s post-hoc test; one of 2 similar experiments shown). **h** Replicates of MΦs used in panel (**g**) were infected using Mtb or BCG and supernatants collected at 18 h were tested for IL-2 and antigen presentation (**p < 0.009; *t* test*)*. **i** PBMC-derived macrophages from healthy donors, household contacts, and TB patients (TB) were treated or not treated with Rapamycin (10 µM) followed by infection with Mtb and CFU counts on day 3 in vitro. **j** Washed Mtb-infected MΦs (as in panel *i*) were overlaid with F9A6-CD4 T cells for antigen presentation with or without Rapamycin and IL-2 assay. Horizontal bars indicate median (interquartile range) for IL-2 (non-normal distribution) or mean (SD) for CFUs (*p < 0.05; **p < 0.001. Wilcoxon paired ranked signed test, and Kruskal-Wallis test). **k** Healthy adult donor MΦs from the TB endemic area differentiated into M1-, M2-, or M0-MΦs were infected with Mtb followed by CFU counts on day 3 (**p < 0.01; Kruskal–Wallis test). **l** Five donor MΦs from each group of panel *k* were treated with siRNA vs. beclin1 or its scrambled control, followed by infection with Mtb and CFU counts on day 3 (**p < 0.009, Kruskal-Wallis test; triplicate wells per donor; one of 2 similar experiments shown). All Mtb CFU experiments used MOI of 1.

**Fig. 5:**
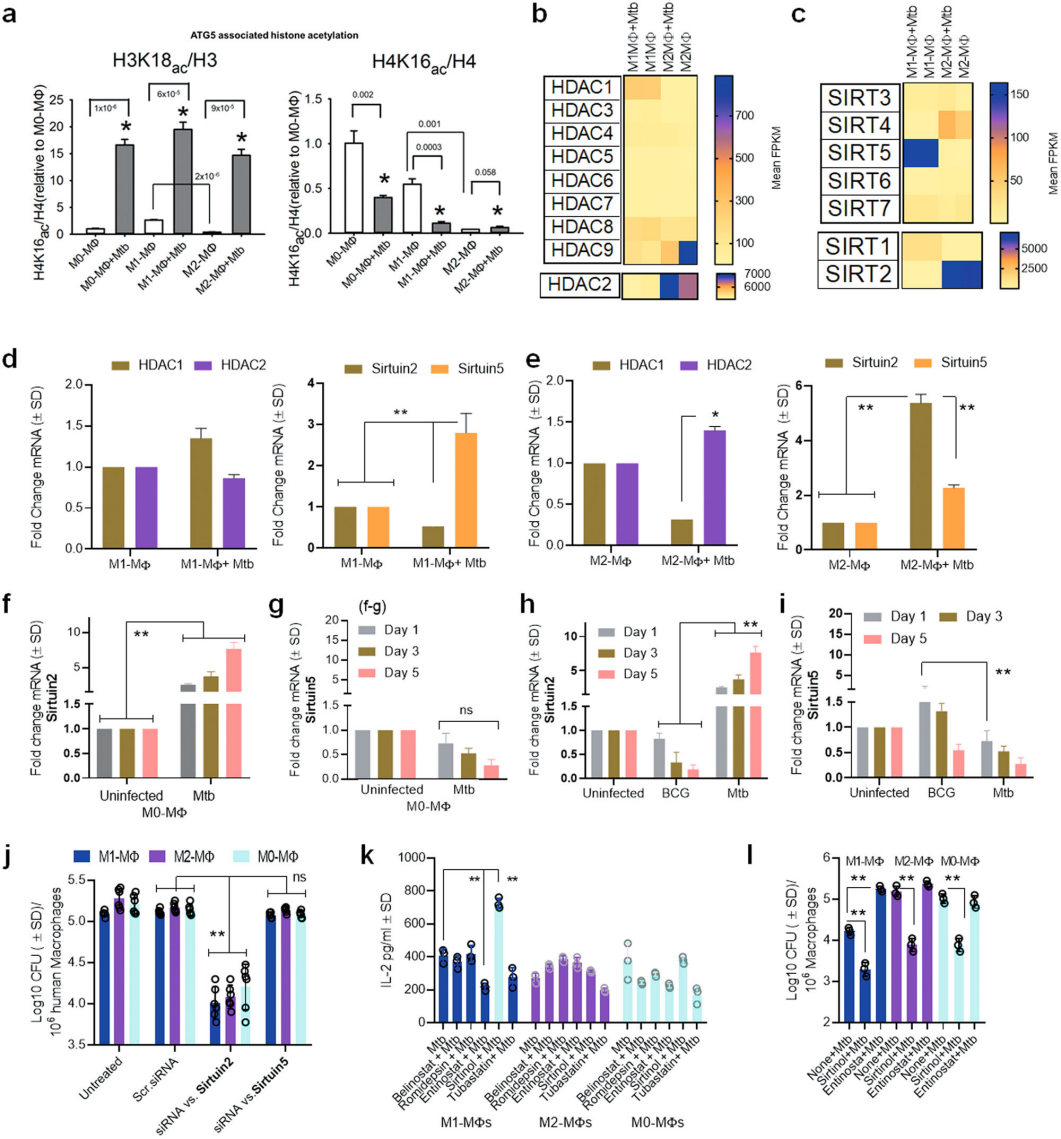
Mtb-infected human M1- and M2-MФs show differential epigenetic programming affecting autophagy through histone acetylation of *ATG5*. **a** H3K18 acetylation and H4K16 acetylation on the promoter of *ATG5* in M0, M1-, and M2-MΦs and their Mtb-infected counterparts (one of 2 similar experiments shown; *p* using one-way ANOVA). **b**, **c** RNAseq gene expression of histone deacetylases (HDACs) and Sirtuins in naïve or Mtb-infected M1-, and M2-MΦs at 18 h. Transcripts are shown as FPKMs (fragments per kilobase per million reads) for duplicate samples. **d** QPCR analysis of mRNA for HDAC and Sirtuins indicated in M1-MΦs at 18 h (**p < 0.01 *t* test; *n* = 2). **e** QPCR analysis of mRNA for HDAC and Sirtuins in M2-MΦs at 18 h (*, **p < 0.01 *t* test; *n* = 2). Densitometry of proteins using western blot shown in Supplementary Fig. 15. **f**, **g** Mtb-infected M0-MΦs were incubated for days indicated and mRNA of Srtuin2 and Sirtuin5 quantitated using qPCR (**p < 0.01 *t* test; *n* = 2). **h**, **i** M0-MΦs were infected with either BCG vaccine or Mtb followed by QPCR analysis of mRNA of Sirtuin2 and Sirtuin5 on indicated days (**p < 0.01 *t* test; *n* = 2). **j** MΦs were treated with siRNA vs. Sirtuins or their scrambled control followed by Mtb CFU assays on day 3 (**p < 0.006, one-way ANOVA with Tukey’s posttest; one of 2 similar experiments shown). **k** Mtb-infected M1- and M2-MΦs were incubated with HDAC inhibitors (Belinostat; Romidepsin; Entinostat; Tubastatin; 20 µM each) and the Sirtuin*-2* inhibitor sirtinol (130 µM), followed by antigen presentation using HLA-DR1-specific F9A6-CD4 T cells (**p < 0.007, *t* test; one of 2 similar experiments shown). **l** MΦs were incubated with Sirtuin2 specific inhibitor sirtinol (130 µM), or the HDAC inhibitor Entinostat (20 µM), followed by Mtb infection and CFU counts on day 5 maintaining >90% viability of MΦs (**p < 0.009, one-way ANOVA with Tukey’s posttest). For all panels one of 2 similar experiments shown). All Mtb or BCG CFU experiments used MOI of 1.

Given the sequential nature of the autophagy cascade, we did not expect to find *ATG* proteins at comparable levels in Mtb-infected M1- and M2-MФ lysates at 18 h post-infection. Western blot analysis of these MФs indicated that Mtb-infected M1- and M2-MФs had higher levels of *ATG proteins* (1, 5, 7, 12, 16L) compared to M2 (supplemental methods, protein simple densitometry*)*. However, we noted that M2-MФs also showed enhanced transcripts for some *ATGs* compared to M0-MФs, although their autophagy-dependent degradation of Mtb was reduced (Fig. 1k**)**. We provide four explanations to account for this interesting difference. First, besides *ATG*s, including LC3 *(ATG8)* (Fig. 3c), autophagosome formation and autophagolysosome fusion requires *SQSTM1* (*also known as. p62*) (Fig. 3f)*, RAB7*, and *LAMP1* (Figs. 1n; 3c; 4a, b), which were highly expressed in Mtb-infected M1-MФs^36^. Secondly, *LC3*, *RAB7*, and *LAMP1* showed stronger colocalization with *rfp*Mtb phagosomes of the M1-MФs indicating autophagic flux (Figs. 1n; 4a, b). Third, when single cells of uninfected M1- and M2-MФs were analyzed for proteins using MILO (methods), *ATG5* and *ATG7*, two proteins essential for macroautophagy, were abundant in M1-MФs compared to M2-MФs (Fig. 3g). Fourth, Mtb evades lysosomal fusion and interferes with autophagy^30^. In addition, we describe below an epigenetic modification through which Mtb differentially affects autophagy in M2- vs. M1-MФs (Fig. 5).

We next sought to define additional functional correlates of gene expression. Because Mtb degrades in APLs producing antigenic peptides, we knocked down selected *ATG*s in Mtb-infected M1, M2-, and M0-MФs using siRNA duplexes (Fig. 3h). We assessed changes in Mtb proliferation using CFU assays and performed a parallel evaluation assessing their ability to present an Ag85B-derived epitope to an F9A6 human HLA-DR1-specific CD4 T cell hybridoma. siRNA knockdown of *ATG5*, *7*, *12*, *16L*, and *Ulk1-ATG1* enhanced the CFU counts of Mtb in M1-MФs but not M2-MФs (Fig. 3i). Notably, autophagy knockdown also led to a decrease in antigen presentation by Mtb-infected M1-but not M2- or M0-MФs (Fig. 3j), indicating a reduced ability of the latter to process and present antigens. These data validate our findings in BCG and Mtb-infected mouse DCs and MФs, where we demonstrated that autophagy plays a major role during Ag85B-p25-mediated T cell activation^29^. Though lysosome mediated Mtb degradation is associated with increased antigen presentation, we note that Mtb can reduce antigen presentation through other mechanisms. For example, Mtb can downregulate MHC-II, interfering with antigen presentation^37^. Intriguingly, Mtb-infected M1-MФs showed increases in antigen-processing components, including MHC-II, vATPase, and cathepsin transcripts (Supplementary Figs. 9, 10). These data indicate that the lysosomes of Mtb-infected M1-MФs are more degradative relative to those of Mtb-infected M2-MФs. Since lysosomal degradation of Mtb enhances the immunogenicity of M1-MФs we analyzed this issue as follows.

### Mtb-infected human M1- and M2-MΦs differentially express enzymes regulating autophagy-depedent antigenic epitope presentation

Mtb-infected M1-MФs expressed multiple *RAB* GTPases compared to M2-MФs (Figs. 3b, 4a). *RAB7* was elevated in M1-MФs during RNAseq analysis (Fig. 4a) and enriched on the *rfp*Mtb phagosomes of M1-MФs (Fig. 4b**;** Supplementary Fig. 3c). Lysosomal marker LAMP1 was also enriched on *rfp*Mtb phagosomes of M1-MФs indicating autophagic flux (Fig. 1m, n). Importantly, siRNA knockdown of *RAB7* led to reduced antigen presentation only among Mtb-infected M1-MФs (Fig. 4c). These data support the notion that *RAB7* regulates lysosomal fusion and is associated with sorting of MHC-II molecules into MIIC compartments for antigen presentation^38,39^.

Mtb-infected M1-MФs expressed transcripts for multiple cathepsins (*CTSA, CTSZ, CTSH, CTSC, CTSL1, CTSS, and CTSB*) compared to M2-MФs (Fig. 4d, e). Even uninfected M1-MФs demonstrated increased cathepsin expression, with *CTSA, CTSZ*, and *CTSH* expressed only by M1-MФs. Because many of the specific antibodies for human cathepsins were not available, we treated Mtb-infected MФs with enzyme-specific or pan-specific cathepsin inhibitors and assessed antigen presentation using an overlay with the F9A6-CD4 T cell line specific for Mtb Ag85B epitope^40^. Cathepsin inhibition markedly decreased antigen presentation by Mtb-infected M1-MФs compared to both M2- and M0-MФs (Fig. 4f). Though all of our tested cathepsin inhibitors reduced antigen presentation in M1-MФs, only pan-specific inhibitors (E64, NALLM) or the *CTSK-*specific inhibitor, balicatib, had inhibitory effects in Mtb-infected M2-MФs. Because CTSK is not a major contributing enzyme during antigen processing, this finding suggested that cathepsin-dependent antigen processing in M2-and M0-MФs is weak. In support of this, a *Clusterprofiler* pathway analysis revealed an upregulation of multiple genes controlling antigen processing in Mtb-infected M1-MФs compared to M2-MФs (Supplementary Fig. 9) and that lysosomes of Mtb-infected M1-MФs were enriched for glycosidases, lipases, and sulfatases in addition to cathepsins (Supplementary Fig. 10). To determine whether bactericidal function is associated with antigen presentation, lysates of MФs treated with cathepsin inhibitors were plated for Mtb CFU counts and replicates tested concurrently for antigen presentation at 18 h. Strikingly, cathepsin inhibition enhanced the survival of Mtb in M1- and M0-MФs but not M2-MФs (Fig. 4g). Because cathepsin inhibition reduced antigen presentation, and enhanced Mtb growth (Fig. 4g, h), we propose that increased levels of lysosomal cathepins is a feature of Mtb-infected M1-MФs compared to M2- or M0-MФs (Supplementary Fig. 9).

After confirming the mechanisms underlying the differential immunogenicity of human MФ phenotypes ex vivo (Fig. 4a–h), we sought to further characterize MФ heterogeneity in vivo during human TB. PBMC-derived MФs of household contacts (defined by their close contact with TB patients), TB patients from the US-Mexico border zone, and healthy donors were tested for their ability to kill Mtb ex vivo in the presence or absence of Rapamycin-induced autophagy in MФs. The bactericidal competency of MФs differed ex vivo even without added cytokines or Rapamycin (Fig. 4i). We note here that unlike M1- and M2-MФs differentiated in vitro, (Fig. 1) PBMC-derived populations likely contained a spectrum of M1- and M2 -phenotypes; therefore, we expressed CFU counts using a linear scale. MФs of healthy controls and contacts degraded Mtb more efficiently than MФs of TB patients, though Rapamycin enhanced bactericidal activity in all groups. To determine whether heterogeneity in bactericidal competency also affected the immunogenicity of PBMC-derived MФs, we further tested their ability to present an Ag85B-derived epitope to F9A6-CD4 T cells. MФs better able to kill Mtb (Fig. 4i) showed an increased present antigen presentation (Fig. 4j). This finding validates our previous report that Rapamycin enhances the immunogenicity of mouse antigen-presenting cells in vitro and in vivo^29,41^ and is consistent with the observation that human monocyte-derived Langerhans DC-mediated antigen presentation is linked to autophagy-dependent degradation of mycobacteria^42^. Notably, some PBMCs may have derived from non-HLA-DR1 subjects and yielded false negatives, although Rapamycin enhanced antigen presentation among all groups of MФs. Because we were not able to purify M1- and M2- phenotypes from TB patients, we once again differentiated M1- and M2-MФs from healthy donors from the same area. These MФs also exhibited differential control over Mtb proliferation; Fig. 4k illustrates CFU counts day 3 post-infection. Furthermore, siRNA knockdown of beclin1/*ATG6* enhanced Mtb proliferation in M1- and M0-MФs but not M2-MФs (Fig. 4l). These data are consistent with the previous observation that PBMCs derived from LTBI donors secreting high levels of IFN-γ upon in vitro activation with Mtb antigen (“high responders”) are of LC3 high phenotype, whereas PBMCs of “low responders” show decreased LC3 levels^43^. Thus, MФ heterogeneity contributes to bactericidal competency and T cell activation during human TB in PBMCs.

Together, these data demonstrate that the lysosomal environment of M1-MФs is strongly degradative for Mtb and autophagy plays an important role during the degradation and processing of Mtb antigens and T cell activation^44–47^. This assumes importance because, classical M1- but not non-classical M2-MФs can differentiate into monocyte-derived dendritic cells, which are also potent human antigen-presenting cells^48^. A relative dominance of M1-MФs during TB can therefore lead to both increased degradation of Mtb and improved T cell activation.

### Mtb infection affects autophagy in human M1- and M2-MФs via epigenetic alteration of ATG5

Because Mtb secretes lysine acetylases, we hypothesized that Mtb modulates autophagy in MΦs through acetylation of *ATG*-associated histones^49^ similar to its ability to enhance IL-10 from naïve MΦs^50^. ChIP-qPCR analysis indicated that, in non-infected and Mtb-infected MΦs, both H3K18 and H4K16 acetylation of the chromatin associated with *ATG5* promoter were higher in M1-MΦs than in M2-MΦs (Fig. 5a). Thus, hyperacetylation of histones correlated with a higher level of autophagy in Mtb-infected M1-MΦs and hypoacetylation correlated with a lower level of autophagy in Mtb-infected M2-MΦs. This is consistent with the observation of Fullgrabe et al, that H4K16 deacetylation is associated with the downregulation of autophagy-related genes^51^ and that Rapamycin decreases histone acetyltransferase hMOF levels facilitating acetylation of *ATGs* and induction of autophagy^51^. Because M0-, M1- and M2-MΦs showed differential acetylation of H3K18 vs.H3K16 after Mtb infection, we propose that Mtb interferes differentially with H3 and H4 acetylation of *ATG5* presumably through lysine acetylases or deacetylases^50^.

To understand the regulatory mechanisms underlying histone acetylation, we analyzed NAD^+^-independent histone deacetylase (HDAC) and NAD^+^-dependent histone deacetylase (Sirtuins) gene expression^52^. Unexpectedly, we found a striking difference in the expression of HDACs. M1- and M2-MΦs both expressed HDACs and Sirtuins. Whereas, Mtb-infected M1-MΦs strongly expressed Sirtuin5, HDAC2 and Sirtuin2 were better expressed by M2-MΦs (Fig. 5b, c). qPCR (Fig. 5d, e) and western blot studies validated gene expression levels for HDAC1/2 and Sirtuin2/5 (densitometry shown in Supplementary Fig. 15). Notably, Mtb infection induced a progressive increase in Sirtuin2 expression in M0-MΦs (Fig. 5f, g). In additional experiments, we found that only virulent Mtb was able to induce Sirtuin2, whereas avirulent BCG vaccine was better at inducing Sirtuin5 but not Sirtuin2 (Fig. 5h, i). These data are consistent with the report that Sirtuin2 is a target for immunotherapy of TB using mice^53^.

Because M2-MΦs had enhanced deacetylase activity, we sought to verify the functionality of HDACs and Sirtuins in relation to autophagy. We knocked down *Sirtuin2* and *Sirtuin5* using siRNA duplexes followed by growth assay. *Sirtuin2* but not *Sirtuin5* knockdown led to an increase in the ability of all three phenotypes of MΦs ((Fig. 5j). Next, we treated MΦs with pharmacological inhibitors of class I and II HDACs or with sirtinol, a dose-dependent specific inhibitor of Sirtuin2, before Mtb infection followed by assays for antigen presentation and Mtb proliferation. Entinostat, which inhibits class I HDAC1 and HDAC3, reduced antigen presentation by Mtb-infected M1- but not M2-MΦs (Fig. 5k). Paradoxically, inhibition of Sirtuin2 enhanced antigen presentation in Mtb-infected M1- but not M2- or M0-MΦs. As Entinostat likely reduces global histone acetylation (especially H3K18 acetylation), and sirtinol preferentially reduces H4K16 acetylation^54–56^, our data are consistent with the acetylation status of the histones of the *ATG5* promoter related to autophagy. Figure 5l shows that Sirtuin2 blockade using sirtinol decreased Mtb survival in M1-, M2-, and M0-MФs. Sirtuin2 is therefore a negative regulator of autophagy and its pharmacological targeting can improve the bactericidal function of M2-MФs. Importantly, Entinostat, which is a broad-spectrum HDAC inhibitor enhanced Mtb proliferation in M1- but not M2- or M0-MФs, underscoring the importance of H3K18 and H3K16 acetylation and autophagy.

Among the HDAC inhibitors we tested, Belinostat, Romidepsin (HDAC1 and 2), and Tubastatin (HDAC6) inhibited acetylation at H3 and H4 using cancer cells but did not affect the ability of Mtb-infected MФs to present antigen to CD4 T cells (Fig. 5i). Sirtuins regulate autophagy^57^ and both Sirtuin1 and Sirtuin3 seem to control mycobacterial proliferation in mice through autophagy^58^. Because Sirtuin5 and Sirtuin2/HDAC2 were upregulated in human M1- and M2-MФs respectively, we propose that human and mouse MФs differ in their regulation of autophagy following Mtb infection. Importantly, enhanced Sirtuin2/HDAC2 deacetylases in M2-MΦs explain in part why their autophagy is reduced despite increased gene expression of some *ATGs* (Fig. 3). Of note, Sirtuin5 is a mitochondrial protein deacetylase, suggesting that acetylation of mitochondrial proteins may also play a role in metabolism and autophagy in M1-MФs. Thus, in addition to IFN-γ, which drives M1-MΦs towards increased autophagy, Mtb-induced epigenetic modifications in ATG5-associated histones can also alter autophagy in M1- vs. M2-MΦs to affect Mtb survival. Notably, Mtb differed from BCG vaccine in selectively inducing Sirtuin2 in naïve MΦs suggesting pathogen dependent modulation.

### Mtb infection alters innate immunity regulating gene (Inregs) expression in human M1- and M2-MФs to affect anti-mycobacterial immunity

Mtb-induced transcriptomic (mRNA) signatures have been analyzed most often in PBMCs and occasionally in bronchoalveolar lavage-derived cells. A meta-analysis of eight studies showed that up to 55 different genes or gene clusters were differentially expressed in the PBMCs of TB patients versus LTBI^59,60^. However, no gene signature was found uniformly in all studies and only 10 genes or clusters were found in more than two studies (Supplementary Fig. 11). Because these studies analyzed unfractionated cells containing a mixture of immune cells including T cells, neutrophils, DCs, and MФs, we hypothesized that detection of M1- and M2-MФ-specific biomarkers associated with bactericidal function may correlate better with resistance and susceptibility, respectively, to TB.

**Fig. 6:**
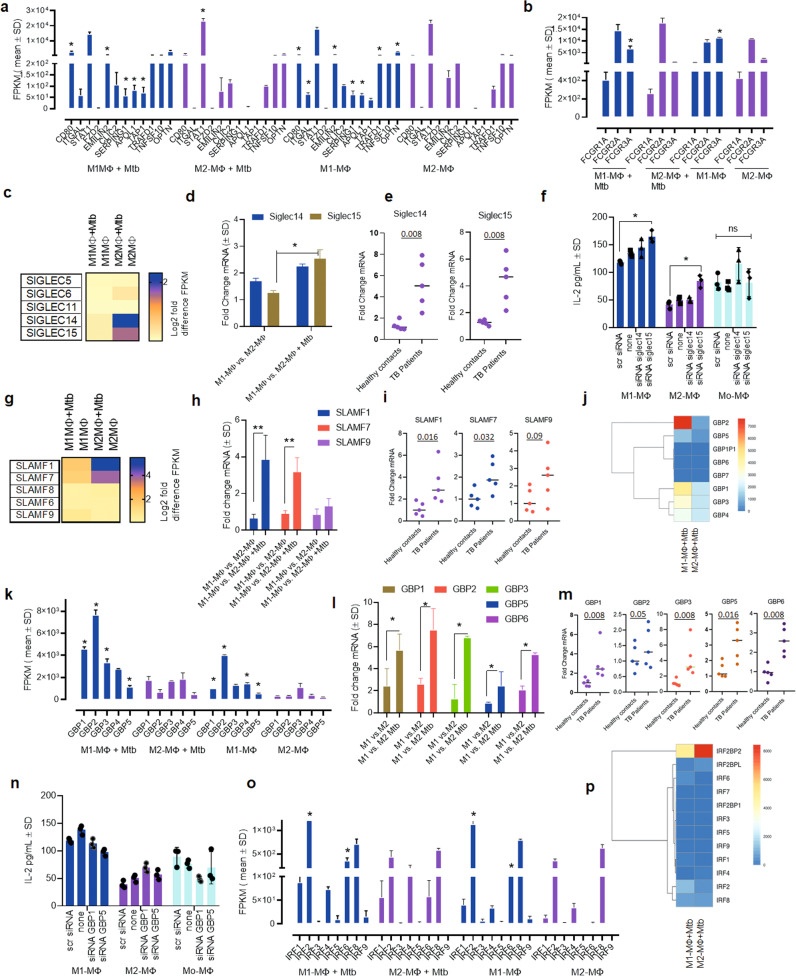
Mtb infection induces *Inregs* in human M1- and M2-MФs associated with anti-mycobacterial immunity. **a**, **b** Mtb-infected and naive M1-, and M2-MΦs show RNAseq-derived differential gene expression for biomarkers at 18 h. Transcripts are shown as FPKMs (fragments per kilobase per million reads. (FPKMs shown; *n* = 2; *p < 0.01 *t* test). **c**–**f** Mtb-infected M1- and M2-MΦs differentially express transcripts (FPKMs) for Sialic Acid Binding Immunoglobin like Lectins (*Siglec*s). **c** RNAseq-derived differential gene expression; Log_2_-fold gene expression. **d** QPCR of mRNA for Siglec-14/15 in Mtb-infected M1- vs. M2-MΦs at 18 h (duplicate sample per assay; 2 similar experiments; *p < 0.01, *t* test). **e** QPCR of mRNA in the PBMCs of children with TB (*n* = 5) and their household contacts at 18 h (n = 5). **f** siRNA knockdown of *Siglec-14/15* in Mtb-infected M1-, M2, and M0-MΦs followed by antigen presentation to F9A6-CD4 T cells. (triplicate wells per assay; 2 similar experiments; *p < 0.009, *t* test). **g**–**i** Differential gene expression for Signaling Lymphocyte Activation Molecule family (*SLAMF*). **g** RNAseq- derived differential gene expression; Log_2_-fold gene expression. **h** QPCR of mRNA in M1- and M2-MΦs at 18 h (duplicate sample per assay; 2 similar experiments; *p < 0.01, *t* test). **i** QPCR in the PBMCs of children with TB and their household contacts. **j**–**m** Differential gene expression for Guanylate-binding proteins (*GBPs*) in M1- and M2-MΦs. **j**, **k** RNAseq- derived differential gene expression (**l**) QPCR of mRNA in Mtb-infected or naïve M1 and M2-MΦs at 18 h (duplicate sample per assay; 2 similar experiments; *p < 0.01, *t* test). **m** QPCR of mRNA in the PBMCs of children with TB and their household contacts (*p < 0.01, *t* test). **n** siRNA knockdown of indicated *GBPs* in M1-, M2, and M0-MΦs followed by antigen presentation to F9A6-CD4 T cells. (triplicate wells per assay; 2 similar experiments; *p < 0.009, *t* test). **o**–**p** RNAseq-derived differential gene expression for Interferon Regulatory Factors (*IRF*s) in Mtb-infected or naïve M1- and M2-MΦs at 18 h. For panels (**a**, **b**, **k**, **o**) FPKMs of Mtb-infected vs. naïve were compared (*< 0.01 *t* test; *n* = 2). All Mtb infections used MOI of 1. Both RNAseq and QPCR analysis done at 18 h post Mtb infection.

We characterized additional differentially expressed transcripts using RNAseq analysis. Because cord blood-derived M1- and M2-MФs are naïve relative to the PBMC-derived MФs of adults, we first determined whether the prenatal MФ-derived M1- and M2-MФs elicited a transcriptional response comparable to that seen in adult MФ-derived M1- and M2-MФs. Transcriptomic analysis of Mtb-infected M1- and M2-MФs in the absence of LPS activation has not yet been reported^23^. Indeed, when M1-MФs programmed with IFN-γ alone or IFN-γ plus LPS were compared, IFN-γ+LPS programmed cells showed elevated gene expression, underscoring the pleiotropic effects of LPS^23^. This is consistent with our report that LPS induces autophagy in MФs through TLR-4 activation^25^. Nonetheless, previous studies using LPS and IFN-γ programmed M1- and IL-4 treated M2-MФs show differential expression of transcripts for *CD80, ITGAL, TNSFS10, FZD2, TRAFD1, STAT1, CLIC2, EMILIN2, OPTN, TAP1, FcG* receptors, and *SERPING1*^23,48^. Interestingly, despite excluding LPS in this study, nearly all earlier reported genes for uninfected M1- and M2-MФs were differentially enriched in cord blood-derived IFN-γ programmed M1- and IL-4 treated M2-MФs (Fig. 6a, b). Because naïve and Mtb-infected human M1-, M2-, and M0-MФs differentially expressed more than 100 genes or gene clusters (Supplementary Fig. 4)^32,61^, including *Inregs* and clusters associated with anti-microbial immunity, we analyzed the functionality of selected *Inregs* using ex vivo QPCR validation of mRNA transcripts in M1-, M2-, and M0-MФs and PBMCs of children with TB and their contacts.

We found that naïve and Mtb-infected M2-MФs differentially expressed Sialic Acid binding Immunoglobulin type Lectins, including *SIGLEC-14*, and *SIGLEC 15* (Fig. 6c–f). QPCR studies showed a superior induction of *SIGLEC-15* mRNA in M1-MФs after Mtb infection (Fig. 6d). Likewise, children with TB had elevated *SIGLEC-14* and *SIGLEC-15* in PBMCs compared to their contacts (Fig. 6e). siRNA knockdown of *SIGLEC15* but not *SIGLEC14* led to increased antigen presentation to T cells in M1- and M2-MФs (Fig. 6f). We conclude that *SIGLEC14/15* are potential biomarkers associated with M1- and M2-MФs that warrants additional studies.

A second gene cluster showing differential expression was Signaling Lymphocyte Activation Molecule Family *SLAMF-1, 7*, *and 9* (Fig. 6g). Our QPCR studies of Mtb-infected M1-MФs showed increased *SLAMF1 and 7* expression ex vivo (Fig. 6h). In a similar manner, PBMCs of children with TB showed elevated SLAMF1/7 and 9 compared to their contacts (Fig. 6i). Preliminary studies showed that siRNA blockade of *SLAMF1,7 and 9* did not affect the ability of MФs to present antigen and need additional studies.

A third gene cluster showing differential expression was Guanylate-Binding Proteins (*GBPs*). *GBP*s are IFN-γ-inducible and Mtb-infected M1-MФs showed enrichment of *GBPs* 1, 2, 3, 5 and 6 (Fig. 6j–l). Intriguingly, these were also enriched in among PBMCs of children with TB compared to controls (Fig. 6m). Because *GBPs* were expressed mostly in Mtb-infected M1-MФs, we propose a defensive role for this gene cluster, although a siRNA knockdown of *GBP1* and 5 did not affect antigen presentation (Fig. 6n). siRNA knockdown of *GBP2, 3 and 6* were not successful. Although additional studies are required to elucidate their function, we note that *GBPs* were not upregulated in Mtb-infected IFN-γ programmed mouse M1-MФs ex vivo^32^, suggesting that *GBPs* likely play a differential regulatory role in mouse vs. human MФs.

The fourth cluster of differentially expressed genes was the interferon regulatory factors (*IRFs*). *IRFs* 2 and 6 were enriched in Mtb-infected M1-MФs compared to Mtb-infected M2-MФs, whereas *IRF8* was expressed at comparable levels (Fig. 6o, p). Of note, *IRF1* and *IRF8* are among the few genes differentially expressed by both Mtb-infected humans (this study) and mouse-derived M1- and M2-MФs^32^.

Taken together, these data indicate that naïve or Mtb-infected cord blood-derived M1- and M2-MΦs not only faithfully reproduce the gene signatures identified earlier in human PBMC transcriptomics, but also reveal gene clusters likely associated with MФ-dependent control of TB. Importantly, the transcriptional responses of human and mouse-derived M1- and M2-MФs differed for *ATGs, RAB GTPases*, cathepsins, *SIGLECs, SLAMF, GBPs*, and *IRFs*, which likely affects the differential control of tuberculosis in mice and humans.

### Inregs expressed by Mtb-infected human M1- and M2-MФs are found in the lymph nodes and macrophages of Mtb-infected neonatal macaques

**Fig. 7:**
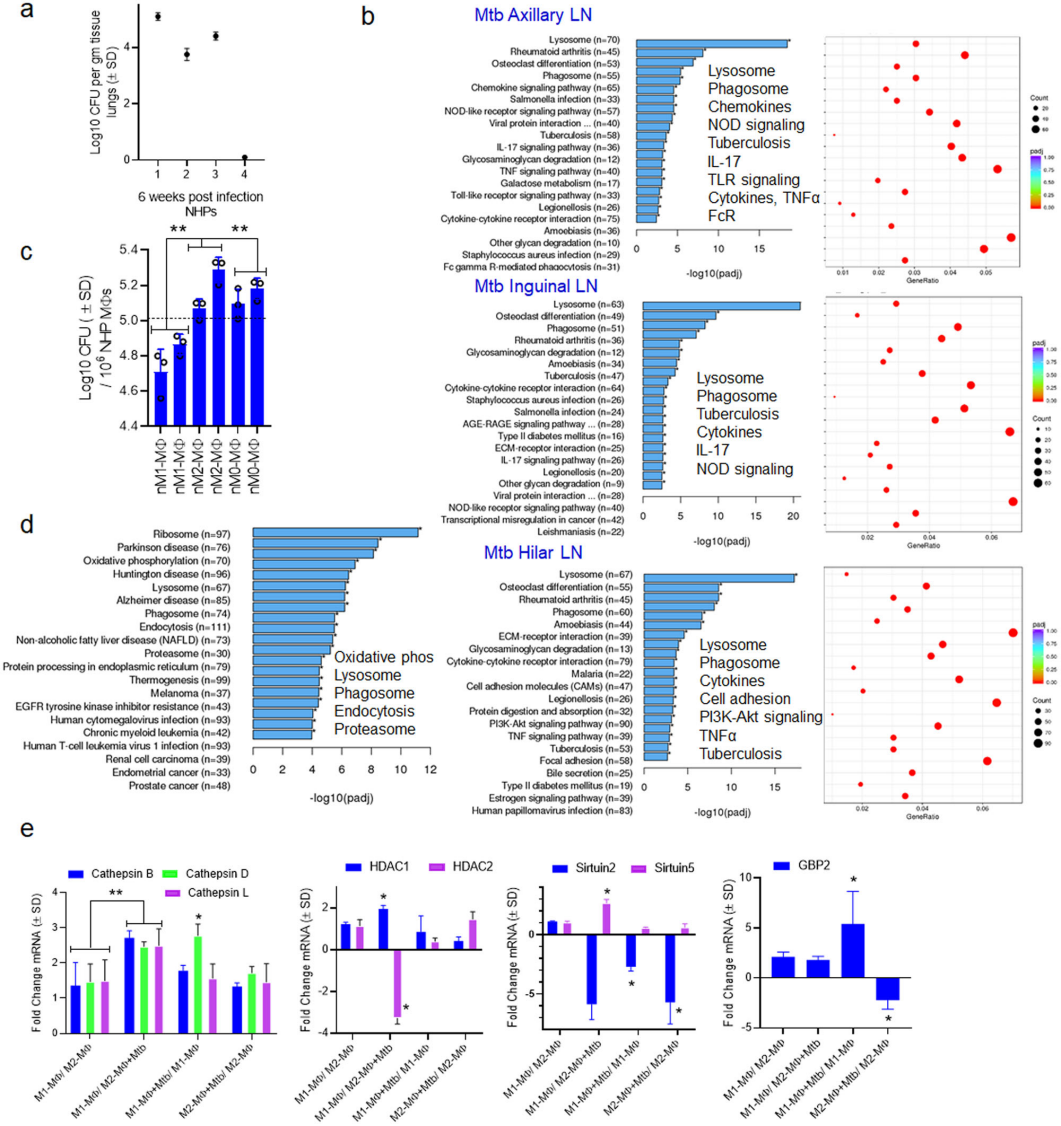
*Inreg clusters* expressed by Mtb-infected human M1- and M2-MФs are found in the lymph nodes and macrophage transcriptome of Mtb-infected neonatal rhesus macaques. **a** Six-week-old rhesus macaques were aerosol-infected with Mtb Erdman strain (25 CFU per animal; *n* = 4) followed by sacrifice at 6 weeks and CFU counts of lungs. **b** lymph nodes (*n* = 2) collected at necropsy from macaques that had comparable Mtb counts in lungs (panel a) were analyzed using RNAseq. Kyoto Encyclopedia of Genes and Genomes profiles of one NHP illustrate the differential gene expression for *Inreg clusters*; *Clusterprofiler* pathway analysis of *Inregs* is illustrated in Supplementary Fig. 12. **c** M1-, M2-, and M0-MΦs were prepared from the bone marrow of naïve macaques (prefix n) and infected with Mtb followed by CFU counts on day 4 (**p < 0.01 one-way ANOVA with Tukey’s post-hoc test; 2 experiments shown). **d** Kyoto Encyclopedia of Genes and Genomes profiles of Mtb-infected M1-MΦs vs. Mtb-infected M2-MΦs show enrichment of genes regulating antigen processing 18 h post-infection. **e** Naïve or Mtb-infected M1-, and M2-MΦs were subjected to QPCR at 18 h post-infection using primers for mRNA of indicated genes which were differentially expressed in their human counterparts (*p < 0.01, *t* test). All ex vivo Mtb CFU experiments used MOI of 1.

To detect *Inregs* during in vivo TB, we infected rhesus macaques with the Erdman strain of Mtb and quantified lung Mtb burden six weeks after infection. Additionally, we isolated MФs from bone marrow collected from naïve NHPs. We performed a transcriptome analysis on lymph nodes collected during necropsy using RNAseq, as they are enriched for MФs^62^ and collection of BAL-derived MФs from infant macaques was not feasible. Figure 7a shows that three of four animals exhibited increased Mtb burden, though one animal had barely detectable Mtb. The lymph nodes from two macaques with high Mtb burden showed an upregulation of gene clusters regulating phagosome and lysosome traffic, NOD-TLR signaling, chemokine-cytokine regulating network, and FcG-R signaling (Fig. 7b). Because these gene clusters were similar to those observed among ex vivo Mtb-infected human M1- and M2-MФs (Fig. 2), we propose that rhesus macaque lymph nodes reproduce human MФ-transcriptional responses to Mtb (Fig. 7b). Unlike ex vivo Mtb-infected human M1- and M2-MФs (Fig. 2), macaque lymph nodes contain many other immune cells. We noted upregulation of additional gene clusters, including the IL-17 network and phosphatidyl-inositol-3 kinase (PI3K)-Akt axis in macaque lymph nodes (Fig. 7b).

To further determine whether human and macaque MФs show comparable gene expression profiles, we differentiated naïve bone marrow-derived MФs into M1-, M2-, and M0-MФ controls and assessed 1) gene expression using RNAseq and 2) their ability to control Mtb growth. Macaque-derived M1-MФs, but not M2- or M0-MФs, inhibited Mtb proliferation (Fig. 7c). Furthermore, a transcriptome analysis of naïve and Mtb-infected NHP M1-, M2-, and M0-MФs showed striking similarities to an analysis of human M1-, M-2, and M0-MФs. For example, ‘antigen processing transcriptome’ genes were similar among NHP and human Mtb-infected M1-MФs (Fig. 2b vs. Fig. 7d). We then validated select RNAseq-identified genes using QPCR. Similar to their human counterparts, NHP M1-MФs upregulated *CTSB, CTSD, CTSL, HDAC1/2, Sirtuin2/5* and *GBP2* (Fig. 7e). Furthermore, MHC-I, MHC-II, and DC-SIGN were upregulated in the regional lymph nodes of Mtb-infected NHPs, whereas cathepsins and other lysosomal enzymes were downregulated (Supplementary Fig. 12). This expression profile is consistent with the development of TB in these animals. Regrettably, we could not maintain NHPs beyond 6 weeks post-challenge to ascertain whether TB could have been controlled by NHPs. With this caveat, we conclude that neonatal macaques display similar MФ heterogeneity to that observed in human MФs.

## Discussion

Most humans exposed to aerosol infection with Mtb do not develop active lung TB. Because both alveolar MΦs (AMΦs) and infiltrating MΦs (IMΦs) are exposed to T cell-derived cytokines during Mtb alveolar infection, we speculated that MΦ subsets may develop differential abilities to resist Mtb infection. An elegant study involving mice and fluorescent Mtb reporters indicated that AMΦs with a fatty acid oxidation cycle less effectively controlled Mtb proliferation, while glycolytically active IMΦs effectively inhibited Mtb^63^. Another study using single-cell RNAseq showed that mouse lungs contain both CD206^−^ (M1-like) and CD206^+^ (M2-like) interstitial MΦs; the latter exerted a suppressive influence through cytokines around the bronchioles, while the former occupied the alveolar space and resembled antigen-presenting cells^64^. Human AMΦs express M1-(CD206^lo^CD86 ^hi^) and M2-like phenotypes (CD206^hi^CD86^lo^) in a steady-state that is altered after alveolar infection^19^. IMΦs are continuously replenished from circulating monocytes. Although TB granulomas contain a variety of immune cells (including M1- and M2-MΦs), few studies have examined M1- and M2- MΦ phenotype-associated gene expression during TB^65–67^. Finally, treatment of diabetics with glybenclamide which regulates potassium and chloride channels, decreased M1-MФs and increased M2-MФs in PBMCs, resulting in an overall decrease in PBMC bactericidal activity against Mtb in vitro^68^. Taken together, these observations strongly support a role for functional M1- and M2-MФ heterogeneity not only at the alveolar and lung level but also in circulating blood.

Coincidentally, transcriptomic studies of PBMCs from TB patients and their contacts showed a limited set of genes associated with either susceptibility or resistance (Supplementary Fig. 11). Although myeloid cell-specific signatures were dominant, a gene expression profile associated with a specific MФ phenotype and function has not been reported for TB.

In this study, we demonstrate that the ability of M1- and M2-MΦs to degrade Mtb is associated with both oxidants and autophagy (Fig. 1). Although independent studies using mouse M1- and M2-MΦs show altered NO and ROS responses or differential LC3 labeling of mycobacteria^69,70^, we found that human M1-MΦs upregulate all three antimycobacterial mechanisms to restrict Mtb (Fig. 1). Though AMФs express M1- and M2- phenotypes^19^, they self-sustain within the alveoli with chemokines facilitating alveolar infiltration by IMΦs and other immune cells. We found that Mtb-infected M1-MΦs secrete several pro-inflammatory cytokines and display increased gene and protein expression of CC- and CXC-type chemokines relative to Mtb-infected M2-MФs (Supplementary Fig. 2). These data suggest that during granuloma formation, M1-MΦ dominance and a pro-inflammatory environment together elicit more robust immune cell recruitment and thereby facilitate an effective TB control response.

Previous studies show that IFN-γ, IL-4, and IL-10 have dramatic effects on Mtb survival^22^. Few studies report the polarization of human M1-MΦs. Among these two studies both used IFN-γ and LPS to drive M1-polarization making it difficult to interpret their bactericidal function since LPS activates autophagy through TLR-4^14,71^ and an LPS-like monophosphoryl lipid A analog is a well characterized adjuvant. Another study used vitamin-D to drive M-1 like differentiation although traditionally M1 phenotype is driven by IFN-γ^20^ and vitamin-D also induces autophagy^72^. This study is therefore the first to rule out LPS-induced pleotropic effects during M1- differentiation. To obtain an in-depth understanding of the signaling mechanisms controlling MΦ function, we performed RNAseq and found that Mtb-infected M1-MΦs expressed a unique transcriptome. Further, gene expression in Mtb-infected human M1- and M2-MΦs was stronger and more diverse than in mouse M1- and M2-MΦs^32^. More than 100 genes were upregulated in Mtb-infected human M1- vs. M2-MΦs (Supplementary Fig. 4) versus less than a dozen in mouse MΦs^32^. Interestingly, Mtb-infected human M1-MΦs expressed a unique ‘antigen processing transcriptome’ (Mtb M1- vs. Mtb M2-MΦs; Fig. 2b), and pathway analysis indicated that Mtb phagosomes were more effectively sorted to APLs for enzyme-mediated degradation and peptide epitope production. This finding is interesting as autophagy has emerged as a major innate immunity mechanism that sorts mycobacteria to APLs for their enzymatic degradation. The peptides epitopes generated are routed for CD4 T cells, whereas autophagy also enhances cross-presentation to CD8 T cells^42,44,46,73,74^. Paradoxically, except for *ATG5-*deficient mice, many *ATG* transgenic mice were not more susceptible to TB^75^. However, we emphasize that, unlike humans, mice are uniformly susceptible to aerosol-induced TB and *ATG*-dependent autophagy does require accessory genes like *RAB* GTPases^76^, and autophagolysosomal mycobacterial degradation requires vATPase and cathepsins.

In this study, we have demonstrated a mechanism by which many TB-exposed humans may resist active disease. Mtb-infected M1-MФs upregulated *ATGs, RAB* GTPases, and autophagy-regulating accessory genes like *MAPLC3IIB (ATG8), LAMP1, galectins* (*LGALS*), and *TRIM* proteins compared to M2-MФs (Fig. 3). siRNA knockdown of beclin1 (*ATG6*) and other *ATGs* increased the growth of Mtb in M1-MФs compared to M2-MФs ex vivo (Figs. 1k, 3i). Together, *LAMP1*, *TRIMs*, and galectins (*LGALS*) regulate various stages of xenophagy and sort mycobacteria to APLs^33,77^. *LGALS*3 was expressed at higher levels by Mtb-infected M1-MФs compared to M2-MФs, but *TRIM16* (a binding partner for *LGALS3*; *aka. Gal3*) was expressed only by Mtb-infected M1-MФs (Fig. 3d, e) and we propose that *LGALS3/8* (*Gal3/8*) and *TRIM38* are biomarkers for Mtb-infected M1-MФs^78^. We conclude that, unlike their mouse counterparts, upon Mtb infection human M1-MΦs strongly upregulate genes associated with their autophagic sorting to APLs.

An interesting consequence of lysosomal degradation of mycobacteria is the peptide epitope-mediated activation of CD4 T cells^79,80^. Mtb and BCG both sequester within immature phagosomes and induction of autophagy delivers them to lysosomes^29,41^. Autophagy also mediates MHC-II and MHC-I-dependent mycobacterial and non-mycobacterial antigen presentation^29,41,44–47^. We found that Mtb-infected M1-MФs upregulated both autophagy and MHC pathway genes and increased expression of key genes for *MHC-I, MHC-II*, vATPase, and multiple lysosomal cathepsins (Fig. 4**;** Supplementary Figs. 9, 10). Increased antigen presentation by Mtb-infected M1-MΦs correlated with *CTSB, CTSL*, and *CTSS*, which are critical during antigen processing and antigen loading into MHC/HLA-DR complexes^81,82^. Further, blockade of *CTSB, CTSL*, and cysteine proteases in MΦs enhanced Mtb proliferation (Fig. 4g), indicating that the bactericidal function of M1- but not M2-MΦs correlates with lysosomal cathepsin function^83–85^. Interestingly, lysosome leakage-derived *CTSB* can also activate the inflammasome during Mtb infection of MΦs, culminating in IL1-β production^86^; Mtb-infected M1-MΦs induced higher levels of the key protective cytokine IL1-β coincident with increased caspase induction (Supplementary Figs. 2, 6). M2-MΦ lysosomes not only lacked an enrichment of cathepsins relative to M1-MΦs but also lacked an enrichment of other hydrolases like glycosidases, sulfatases, and lipases (Supplementary Fig. 10**)**. These data provide a mechanistic explanation for the link between MΦ lysosomal disorders and increased TB susceptibility^84^. Taken together, these results provide new insight into how enhanced lysosomal enzymes may enable M1-MΦs to both kill Mtb and degrade them to produce antigens for T cell activation. Conversely, the results suggest that M2-MΦs show a reduced immunogenicity phenotype due to reduced lysosome function.

A third major mechanistic insight from this study is based on the paradoxical role MΦs play in both degrading intracellular Mtb and acting as a niche for Mtb persistence over decades. Mtb is a unique pathogen that secretes or sheds multiple virulence factors to facilitate immune evasion^87^. For example, Mtb secretes *sapM* phosphatase, which interferes with phagosome-lysosome fusion in MΦs. Indeed, a ∆*sapM* strain of Mtb was attenuated but immunogenic in mice^88,89^. Others have also shown that Mtb evades the autophagy pathway leading to APLs^30,90^. Herein, we demonstrate a regulatory mechanism wherein Mtb infection of M1-MΦs increases *ATG5*-associated histone acetylation^51,91^. We also show that the decreased autophagy phenotype of M2-MΦs is maintained by their elevated expression of HDAC-Sirtuins and they also show a reduced expression of *LC3, RAB7* and *LAMP1* which regulate autophagolysosomal fusion. Importantly, pharmacological activation of autophagy seems feasible for M2-MΦs which can also increase their immunogenicity (Fig. 5). While Everolimus (a purified form of Rapamycin) is being used in combination with anti-TB drugs to augment autophagy during host-directed therapy, others have used HDAC/Sirtuin inhibitors for augmenting host defense^92^. Rapamycin decreases the hMOF acetyltransferase enhancing autophagy and we provided evidence that it enhances degradation of Mtb (Fig. 1)^51^. Intriguingly, the class I HDAC inhibitors trichostatin and SAHA had an unexpected adverse effect on MΦ-mediated Mtb uptake and degradation^93^; our data therefore underscore the importance of understanding the differential expression of HDAC/sirtuins by M1- and M2-MΦs before devising host-directed therapy.

IFN-γ reprograms MΦs for anti-mycobacterial function but can also enhance autophagy^94,95^. Accordingly, low-dose activation followed by resting still left a remarkable imprint on M1-MΦs, which enabled them to respond rigorously to Mtb infection. Even then, Mtb persisted in both M1- and M2-MΦs, although long-term cultures showed a gradual CFU decline in M1 vs. M2-MΦs and enhanced cell death in M2-MΦs. Because of these interactions between host and Mtb- dependent histone modifications, we conclude that Mtb is likely to persist in a non-replicating state in M1- but not M2-MΦs particularly in view of the data that Mtb-infected M1- but not M2-MΦs continue to secrete GM-CSF (Supplementary Fig. 2). We therefore propose that selective regulation of histone acetylation using HDAC and Sirtuin inhibitors in combination with anti-TB drugs may help to both control and eradicate Mtb from MΦs irrespective of their phenotype.

The fourth novel observation from our study is that *Inreg* signatures identified using M1- and M2-MΦ transcriptomes are detectable in PBMCs for children with TB and their contacts. Because M1 and M2-MΦs differentially control Mtb proliferation, we hypothesized that detection of *Inregs* like *SIGLEC*, *SLAMF*, and *GBP* family of genes may help to differentiate between LTBI and TB patients. These *Inregs* showed a striking difference in expression within M1- vs. M2-MΦs ex vivo, and were also able to sufficiently discriminate TB patients from their contacts (Fig. 6). Interestingly, at least two studies report a genetic association between *SIGLEC14/15* and susceptibility to TB^96,97^. *SIGLEC15* is also known as an immunosuppressive checkpoint during cancer immunotherapy^98^ and siRNA knockdown of *SIGLEC15* increased antigen presentation by MΦs (Fig. 6f). In contrast, *SLAMF1/7* members which were enriched in Mtb-infected M1-MΦs (Fig. 6h) are associated with both positive and negative regulation of chemotaxis, autophagy, and plasmacytoid DC function^99–101^. *GBPs* which were associated strongly with M1-MΦs discriminated contacts from TB patients (Fig. 6m) and were earlier identified in 4 of 8 transcriptomic studies on TB patients and their contacts^60^. *GBPs* regulate phagosome maturation, autophagy, mycobacterial antigen processing, and cell-autonomous or innate killing of intracellular pathogens^102–105^. We conclude that M1- and M2-MΦ-associated *Inregs* are more likely to discriminate between TB-resistant and TB-susceptible individuals and are newer targets for diagnosis and immunotherapy of TB.

Finally, to validate gene expression findings from ex vivo cultured and Mtb-infected human MΦs, we developed a neonatal macaque model of TB. Using this model, we show the first in vivo transcriptional profiles for lymph nodes following TB. Although we also analyzed lung transcriptomes, we sought to focus on lymph nodes because they are enriched in both MΦs and DCs and play a major role in regulating immunity to TB^106^. Unlike mice, adult NHPs show a dose-dependent susceptibility to TB, although infant macaques are more susceptible^107–109^. For example, about a third of adult rhesus macaques develop TB after aerosol infection with an Erdman strain of Mtb, whereas we found that three of four neonates showed a significant Mtb burden in the lungs (Fig. 7a).

Our studies suggest a potential reason for the differential susceptibility of macaques to TB. Although the lymph nodes contained multiple types of immune cells, the myeloid regulatory pathways of phagosome/lysosome, NOD/TLR signaling, cytokine-cytokine interactions, cell adhesion, and MAPK were transcriptionally enriched, similar to Mtb-infected M1-MΦs (Fig. 7b vs. Fig. 2b). Paradoxically, Mtb infection upregulated MHC-I and MHC-II genes but downregulated cathepsins and lysosomal hydrolases essential for TB control during LN profiling (Supplementary Fig. 12). Furthermore, NHP bone marrow-derived Mtb-infected M1- and M2-MΦs also displayed differential control over Mtb proliferation (Fig. 7d) and differed in their enrichment of genes associated with the antigen processing transcriptome (Fig. 7e).

In an earlier study, PBMCs from Mtb-infected rhesus and Chinese cynomolgus monkeys displayed differential expression of genes associated with M1- and M2- phenotypes, although they were not examined for their ability to degrade Mtb, and M1- and M2-MΦs were not enriched from these primates to assess molecular mechanisms^110^. As we recently reported that some adult NHPs can still prevent reactivation of TB despite severe T cell loss due to Simian Immunodeficiency Virus^111^, we propose that MΦ heterogeneity may play an important role during TB in macaques. Regrettably, we were unable to follow Mtb infection beyond 6 weeks post-infection, which precluded us from determining whether infant macaques could have controlled TB beyond 6 weeks leading to latency. Nonetheless, our observations argue that NHP MΦs mirror human MΦs in their transcriptional responses to TB. Therefore, infant macaques provide a novel model with which to develop vaccines and determine the role of myeloid vs. lymphoid cell-mediated protection against TB.

In conclusion, we demonstrate that human MФ subsets show a unique pattern of gene expression that enables differential control over TB proliferation in humans and macaques. Some of the phenotype-specific genes are promising targets for vaccine development, diagnostics or therapeutics.

## Methods

### MΦs from cord blood, PBMCs from healthy donors, contacts or tuberculosis patients

All blood samples were collected per approved institutional Institutional review board protocols and included HLA-DR1 positive donors. To correlate gene expression with antigen processing, only HLA-DR1 + PBMCs were used. CD14 magnetic beads (Miltenyi Inc., USA) were used to purify monocytes which were plated in 6 or 24 tissue culture plate wells at a density of 4 × 10*6 and 1 × 10*6 cells per well, respectively. 8-well slide chambers or cover slips received 10*3 cells per chamber for confocal IF studies. CD14 bead purified monocytes were grown in Iscove’s medium with 10% fetal bovine serum and 10 ng/mL GM-CSF for 6 days and then plated in GM-CSF free medium for 24 h before differentiation them into M1- and M2- macrophages. Both heat-inactivated AB serum and fetal bovine serum were concurrently evaluated for phenotype studies and no differences were found. M1- and M2–MΦs were obtained by incubation with IFN-γ (10 ng/mL) or IL-4 (10 ng/mL) respectively for 5 days after which they were rested for 2 days. M0-MФs were not treated with any cytokine. Human TB patients and contacts: These were collected from deidentified, known TB patients and their healthy contacts with approved Institutional review board protocols of Dr. Restrepo from Reynosa, Mexico under a collaboration. Approved Institutional review board protocol HSC-SPH-12-0037. PBMCs collected in trizol from deidentified children with confirmed TB or household contacts from Vietnam were kindly provided by Drs. Nhung Nguyen and Ha Phan under a collaboration with Dr. Graviss. Table 1 provides details of their clinical status.

**Table 1:** Clinical classification of peripherl blood samples from children with tuberculosis and those exposed to tuberculosis.

#	Group	PBMC	DOB	TB contact	History of TB	TB status	Current RX		Gene expert
C01	Control	9/13/2019	1/14/2008	No	No	BPTB	No		Negative
Co2	Control	9/13/2019	9/3/2012	No	No	BPTB	No		Negative
C03	Control	9/13/2019	8/13/2017	No	No	BPTB	No		Negative
C04	Control	9/13/2019	12/31/2017	No	No	BPTB	No		Negative
C05	Control	9/13/2019	9/30/2009	No	No	BPTB	No		Negative
TB01	PediatricTB	9/13/2019	2/12/2019	Yes	No	BPTB + EPTB	Yes	2RHZE	Negative
TB02	PediatricTB	9/13/2019	10/4/2004	Yes	No	BPTB	Yes	2RHZE	Negative
TB03	PediatricTB	9/13/2019	2/15/2012	Yes	No	BPTB + EPTB	Yes	2RHZE	Positive
TB04	PediatricTB	9/13/2019	7/5/2011	Yes	No	EPTB	Yes	2RHZE	
TB05	PediatricTB	9/13/2019	8/24/2005	Yes	No	BPTB	Yes	2RHZE	

*BPTB* broncho-pulmonary TB, *EPTB* extrapulmonary TB, *RHZE* rifampin, isoniazid, pyrazinamide and ethambutol.

### Mtb and BCG infection of MΦs

Methods to prepare Mtb (H37Rv) and BCG (Pasteur) strains for infection have been described earlier (29). MΦs were infected (MOI = 1) using Mtb or BCG for 4 h prepared as follows. M. tuberculosis (H37Rv) (American Type Culture Collection-27294) (Mtb) was grown in BBL™ Middlebrook 7H9 broth with OADC enrichment (BD Biosciences, 211886) at 37 °C and 5% CO_2_. Green-fluorescent-protein-expressing *M. tuberculosis* H37Rv (*gfp*Mtb) and red-fluorescent-protein-expressing M. tuberculosis H37Rv (*rfp*Mtb) were a kind gift from Dr. Malini Madiraju (The University of Texas at Tyler, Tyler, TX). All mycobacterial strains were grown for 7 days in 7H9 broth with (for gfp/rfp strains) or without 25 µg/mL kanamycin, and aliquots containing ~10*8 viable CFUs were frozen for subsequent use. Before use, aliquots were thawed, washed three times in phosphate-buffered saline (×12,000 rpm; 15 min), and sonicated at 4 W with a sonicator (60 Sonic Dismembrator, Fisher Scientific) to prepare a uniform single-cell suspension of Mtb without loss of viability.

### Phagocytic uptake and viability of Mtb or BCG-infected MΦs

Phagocytic uptake of rfpMtb by MΦs was determined by washing to remove extracellular bacilli with medium 4 times, fixation in ice cold methanol followed by microscopic counts of fluorescent bacteria per 500 MΦs in triplicate slide chambers. MΦs treated with pharmacological agents, siRNA or infection were carefully monitored for viability using trypan blue exclusion assay to maintain >90% viability until 7 days post-infection, although depending upon the assay, they were harvested either 18 h, 3, 5 or 7 days post-infection. No signs of apoptosis were observed at MOI = 1 for Mtb or BCG until day 7 post-infection. *Tunel* assays (Sigma Aldrich, Inc) were used to confirm the lack of apoptosis in all cultures and they were routinely tested for LPS and mycoplasma contamination.

### RNAseq analysis

Naïve or Mtb-infected M1-, M2- and M0-MΦs (≥2 × 10 *6 cells/pellet; *n* = 2; numbers matched for all samples) were collected into trizol buffer and snap frozen in liquid-N2. RNASseq analysis, data interpretation and pathway analysis were done by Novogene (USA) as described (Supplemental Fig. 13). RNAseq was done two separate times using trizol samples (*n* = 2). Additional bioinformatic analysis was done in-house by coauthor PK.

### Ex vivo Mtb Ag85B antigen presentation to CD4 T cells

This has been described in detail by us (29) and the original method described by the Harding lab has been extensively used by us and others for in vitro antigen presentation by MΦs (29) Briefly, Mtb-infected MΦs were washed after a 4 h infection and overlaid with the F9A6-CD4 T cell hybridoma (Dr. David Canaday) which recognizes an Ag85B epitope in the context of human HLA-DR1. IL-2 secreted from hybridoma T cells or other cytokines secreted from Mtb-infected MΦs were determined using a sandwich enzyme-linked immunoassay kit (Ebiosciences). Where indicated, the MΦs were pharmacologically blocked with various inhibitors or using siRNA probes (Supplementary figures). Cytotoxicity/viability of macrophage (>90%) was carefully monitored for indicated periods using trypan blue assay.

### Western blot experiments

Six-well tissue culture plates were seeded with M1-, M2- and M0-MΦs. They were infected with Mtb for 4 h (h) at MOI of 1 and then washed three times with phosphate-buffered saline and re-plated in the medium. At different time points, MΦs were washed three times with 1x phosphate-buffered saline, and 200 µL RIPA buffer containing anti-protease mix was added to each well and incubated for 15 min. Lysates were then collected, and protein quantification (bicinchoninic acid, Pierce 23225) was performed. a) For western blot, 25 µg of total protein was loaded per well of Bio-Rad criterion gels and transferred to polyvinylidene fluoride membranes. Antibodies used to probe for proteins using the Bio-Rad apparatus or Protein Simple “Wes” automated capillary electrophoresis are listed in Supplemental Fig. 14 and Methods. Blot polyvinylidene fluoride membranes were then developed using an enhanced chemiluminescent kit or using Wes. Western blot and densitometry analysis data are shown as mean band density normalized relative to GAPDH (*n* = 2). The “Protein Simple” capillary gel electrophoresis with automated protein readings and densitometry was calculated as indicated.

### siRNA knockdown in Mtb-infected or naïve M1-, M2- and M0- MΦs and Mtb CFU assay

The kits for various human siRNAs (mixture of duplexes), were purchased from Origene as listed in Supplementary Tables. MΦs were treated with siRNA and the scrambled control according to the manufacturers’ instructions, and this was followed by addition of Mtb (H37Rv) for 4 h. (MOI of 1). Cells were then lysed, and 10-fold dilutions were plated on 7H11 agar plates for CFU counts, which were read after 21 days of incubation. Details of the CFU assay have been described elsewhere (29). When CFUs were read day 3 after infection, a linear scale was used to express data whereas, day 5 CFUs were determined log scale. Mtb replicates with a generation time of about 18 h. For autophagy induction and effects on Mtb CFUs, a dose range of 1–10 µM of Rapamycin and 50–100 µM of Metformin were used.

### Pharmacological blockade of Cathepsins, HDACs and Sirtuins on the viability of Mtb in M1-, M2- and M0-MΦs

The reagents used to blockade are listed in Supplementary Tables. MΦs were treated with 1 µg/mL of indicated agents followed by a 4-h infection with Mtb, washing and incubation with drugs as before for 3-5 days. On day 3-5, cells were then lysed, and 10-fold dilutions were plated on 7H11 agar plates for CFU counts, which were read after 21 days of incubation. Viability of MΦs was maintained at >90% through the assay period as determined by trypan blue assay.

### Evaluation of autophagosome puncta, lysosomes, and biomarker localization with gfp- or rfpMtb within M1-, M2- and M0- MΦs

M1-, M2- and M0-MΦs were plated in 8-well chamber slides at a density of 10*3 cells per chamber and infected with either *gfp*- or *rfp*Mtb at a MOI of 1. Per established procedures, autophagy was evaluated using at least three criteria. Fixed MΦs were permeabilized and stained using specific validated antibodies against LC3 autophagosomes; RAB7 and LAMP1 autophagolysosomes followed by secondary staining with Alexfluor485 /Alexafluor590 anti-IgG (Jackson Immunoresearch #111-095-003) as described by us (29). Percent *gfp/rfp*Mtb phagosomes colocalizing with antibodies was done as described by us (29). To avoid visual bias, region of interest was measured as described^112^.

### Flow cytometry of M1-, M2- and M0 macrophages

Flow cytometric analysis of M1-, M2-, and M0-MΦs before and after infection with Mtb was done per published procedures. MΦs were stained and analyzed on the Fortessa flow cytometer (Beckton Dickinson), and the data were processed using FlowJo v10 software (Tree Star, Inc.). Fluorochrome-conjugated antibodies used for flow cytometry were as follows: PE-Cy7 anti-human CD68 (BD Biosciences, cat no. 565595; clone: Y1/82A), PE anti-human CD14 (Invitrogen, cat no. 12-0149-42; clone: 61D3), APC anti-human CD206 (BioLegend, cat no. 321110; clone: 15-2), AF700 anti-human CD80 (BD Biosciences, cat no. 561133; clone: L307.4). Dead cells were excluded by using aqua fluorescent reactive dye (Invitrogen, cat no. L34957). GraphPad PRISM used for data analysis. Other antibodies are listed in Supplementary Tables.

### Cytokine and chemokine assays

Cell supernatants were tested using sandwich enzyme-linked immunoassay kits (BioLegend and R&D systems, USA) for various cytokines and chemokines secreted by M1-, M2- and M0-MΦs before and after infection with Mtb.

### NO and ROS assays

M1-, M2- and M0- MΦs were plated in 96-well plates at a density of 1 × 10*3 cells per well in triplicates and infected with Mtb (MOI = 1). Cells were then treated with fluorescent probes for the quantification of NO using diaminofluorescein diacetate (DAF-2 DA), per the manufacturer’s instructions (Enzo Life Sciences, USA). To differentiate whole cell ROS detected using DCFDA from mitochondrial ROS, we used a similar procedure and MitoROS reagent. To express nitrite levels, mouse MΦs were activated using LPS and IFN-γ followed by diaminonathalene assay and fluorescence relative light unitss measured correlated with Greiss reagent measurements. Nitrite levels in human MΦs were in nM levels unlike µM for mouse MΦs. Quantification of NO release was done by calculating the fluorescence emitted over time using an ex485 nm/em515 nm and plotting AFUs (± SD) against time using Ascent fluoroscan software version 2.6. These assays were described by us earlier (27). Likewise, DCFDA was used to detect ROS (Supplementary Fig. 3). In addition, iNOS mRNA and protein were quantitated using qPCR and MILO blots as described in methods and Supplementary Table 2.

### Dual cross-link chromatin immunoprecipitation-Quantitative real time genomic PCR (xChIP-qPCR) for ATGs

Cells (4 × 10*6 to 6 × 10*6 per 100-mm dish) were washed twice with phosphate-buffered saline. Protein-protein cross-linking was first performed with disuccinimidyl glutarate (2 mM, Pierce), followed by protein-DNA cross-linking with formaldehyde. Equal amounts of sheared chromatin were immunoprecipitated overnight at 4 °C with 4 μg of anti-rabbit primary antibodies of H3, H3K18ac, H4, and H4K16ac (Cat#39763, 39587, 61521, and 39167, Active Motif, Carlsbad, CA) in ChIP dilution buffer. Anti-rabbit IgG was used as the negative control. Immunoprecipitates were collected with 40 μL protein A magnetic beads (Dynal Inc.), washed, and eluted in 250 μL elution buffer for 15 min at room temperature. Samples were de-cross-linked in 0.2 M NaCl at 65 °C for 2 h. The precipitated DNA was phenol/chloroform-extracted, precipitated with 100% ethanol and dried. Gene enrichment in xChIP was determined by Q-gPCR. Specific regions for the ATG5 promoter: Sense, (−1900) 50-CAGGGTCTCTCTCTGTTACC-30 (−1881), and Antisense, (−1669) 50-CCCAAAGTGCTGGGATTACA-30 (−1651). Standard curves were generated using a dilution series of genomic DNA (from 1 ng to 100 ng). The fold change of DNA in each immunoprecipitate was determined by normalizing the absolute amount to the input DNA reference and calculating the fold change relative to that amount in M0-MΦ cells. All data of Quantitative-genomic PCR shown in present study are the mean ± SD from three independent experiments.

### Neonatal rhesus macaque model of tuberculosis

Six-week-old rhesus macaques were infected using 25 CFU per animal of Mtb Erdman using Institutional Animal Care and Use Committee approved procedures (# 1655-MM, Texas Biomedical Center) described in detail earlier^107^. They were sacrificed at 6 weeks post-infection, followed by necropsy for bacterial burden of lungs, and collection of left and right axillary, inguinal and hilar lymph nodes. Two sets of each type of lymph nodes from 2 macaques were suspended in trizol and analyzed for RNAseq by Novogene Inc. USA. For pathway analysis, matching lymph nodes from naïve macaques were concurrently analyzed. Bone marrow-derived CD14 bead purified, MΦs were differentiated as described for human MΦs into M1-, M2- and M0- MΦs and then analyzed for their ability to kill Mtb ex vivo; RNAseq analysis was done and QPCR analysis for select genes as described for human MΦs.

### Single-cell protein analysis using MILO instrument

Single-cell western blot assays were performed using the Protein Simple MILO platform with the standard single-cell west (ScWest) kit according to manufacturer’s protocol. ScWest chips were rehydrated and loaded with cells at a concentration of 100,000 cells/mL suspension buffer. 100,000 macrophages loaded/chip, with 21–22 min settling time, 5 s lysis, and 60 s separation time. Multiplet/doublet capture rate in scWest chip microwells was determined with light microscopy (~1.3%, established from >1000 microwells). scWest chips were lysed for 10 s and instantly followed by electrophoresis for 80 s at 240 V. UV light exposure provided for total 4 min to get protein immobilized. scWest chips were sequentially probed with primary for 2 h and secondary antibodies for 1 h. Primary antibodies used were anti-mouse ATG7 (1:10, MAB6608; R&D), anti-rabbit ATG5 (1:10, 12994 s; Cell Signaling) and anti-rabbit GAPDH (1:10, 5174 s; Cell Signaling). Secondary antibodies used were goat anti-rabbit Alexa Fluor 647 (ATG7, 1:20), goat anti-mouse Alexa Fluor 555 (ATG5, 1:20) and goat anti-mouse/rabbit Alexa Fluor 488 (GAPDH, 1:20). Slides were washed, centrifuge-dried, and scanned with Innopsys Microarray Scanner. Data was analyzed using Scout software (Protein Simple) and ImageJ (NIH). Blot profiles for Figures are also indicated in the supplemental information.

### Statistics and reproducibility

P values for CFU counts in macrophages were determined using 1 or 2-way ANOVA with Tukey’s post-hoc test (GraphPad PRISM software). Fragments Per Kilobase of transcript per Million mapped reads (FPKMs) within groups (Mb-infected versus naïve) were analyzed using Student’s two tailed *t* test. Data from human donors and tuberculosis patients: within group differences were established by Wilcoxon paired ranked signed test; between group differences (household contact versus TB) were determined by Kruskal–Wallis test. All experiments were done 2–3 times with biological triplicates with similar results and representative experiments are shown; when duplicates were used, they are indicated.

### Reporting summary

Further information on research design is available in the Nature Research Reporting Summary linked to this article.

## Supplementary information

Supplementary Information

Description of Additional Supplementary Files

Supplementary Data 1

Reporting Summary

## Data Availability

The RNAseq raw data will be uploaded into the NCBI, NIH database; supported by NIH 1RO1 AI-122070 (C.J.). All other data are available from the corresponding author (or other sources, as applicable) on reasonable request. A source data table has been included as Supplementary Data 1.
