# Supplementary material for: Human M1 macrophages express unique innate immune response genes after mycobacterial infection to defend against tuberculosis
Source: Commun Biol. 2022 May 19;5:480. doi: 10.1038/s42003-022-03387-9 (PMC9119986; doi:10.1038/s42003-022-03387-9)

Human M1 macrophages express unique innate immune response genes after mycobacterial infection to defend against tuberculosis.

Arshad Khan[1*], Kangling Zhang[2*], Vipul K. Singh[1*], Abhishek Mishra[1], Priyanka Kachroo[1], Tian Bing[2], Jong Hak Won[3], Arunmani Mani[3], Ramesha Papanna[3], Lovepreet K. Mann[3], Eder Ledezma-Campos[4], Genesis Aguillon-Duran[4], David H. Canaday[5], Sunil A. David[6], Blanca I. Restrepo[7], Nhung Nguyen Viet[8], Ha Phan[9], Edward A. Graviss[1], James M. Musser[1], Deepak Kaushal[10], Marie Claire Gauduin[10], and Chinnaswamy Jagannath[1]

[1]*Dept. of Pathology and Genomic Medicine, Houston Methodist Research Institute, Weill-Cornell Medicine, Houston, TX*

[2]*Dept. of Pharmacology and Toxicology, University of Texas Medical Branch, Galveston, TX*

[3]*Dept. of Obstetrics, Gynecology and Reproductive Sciences, UTHSC, Houston, TX, USA*

[4]*Centro Regional de TB, Secretaría de Salud de Tamaulipas, Reynosa, Mexico*

[5]*Division of Infectious Disease, Case Western Reserve University, Cleveland VA, OH, USA*

[6]*Virovax, LLC, Adjuvant Division, Lawrence, Kansas, USA.*

[7]UT *School of Public Health, Brownsville, and STDOI, UT Rio Grande Valley, TX. USA*

[8]*National Lung Hospital, Ha Noi, Vietnam*

[9]*Center for Promotion of Advancement of Society, Ha Noi, Vietnam*

[10]*Southwest National Primate Research Center, Texas Biomedical Research Institute, San Antonio, TX, USA.*

**Contributed equally to the manuscript.*

Correspondence to: Chinnaswamy Jagannath. Ph.D.

Professor, Dept. of Pathology and Genomic Medicine, Center for Molecular and Translational Human Infectious Diseases Research. Houston Methodist Research Institute, Weill-Cornell School of Medicine, Houston, TX 77030

Email: cjagannath@houstonmethodist.org

Phone: 713-459-7301 Fax: 713-441-7925

Competing interests: The authors declare no conflict of interest and certify that all studies were conducted under approved institutional Health Science Center and Institutional review board protocols.

Keywords: *human macrophages, Mycobacterium tuberculosis, BCG, M1-, M2-, macrophage phenotype, autophagy, IFN-γ, IL-4, GBP, IRF, SIGLEC, SLAMF, ATG, RAB GTPASE, HDAC, Sirtuin, cathepsin, Rapamycin, antigen presentation, epigenetics, histone acetylation, transcriptomics, neonatal rhesus macaques*

Supplemental Figure-1: Gating strategy for M1 and M2 macrophages before and after infection using *M. tuberculosis (Mtb)*. Related to Figures 1a and 1b.

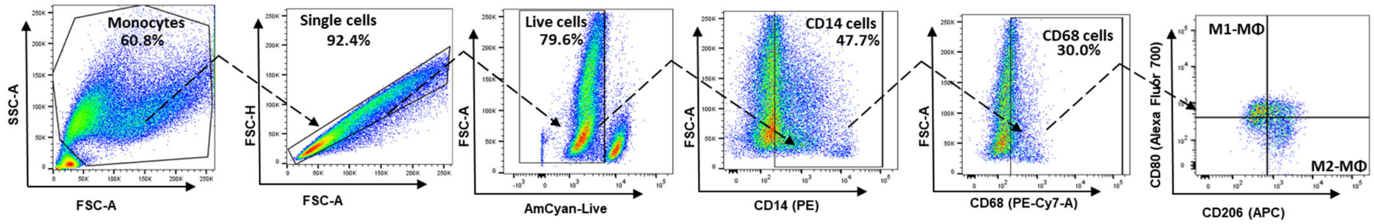

M1-Mϕ gating: Monocytes +ve , Single cells, live cells, CD14⁺ cells, CD68⁺ cells, CD80⁺CD206⁻

M2-Mϕ gating : Monocytes +ve , single cells, live cells, CD14⁺ cells, CD68⁺ cells, CD206⁺CD80⁻

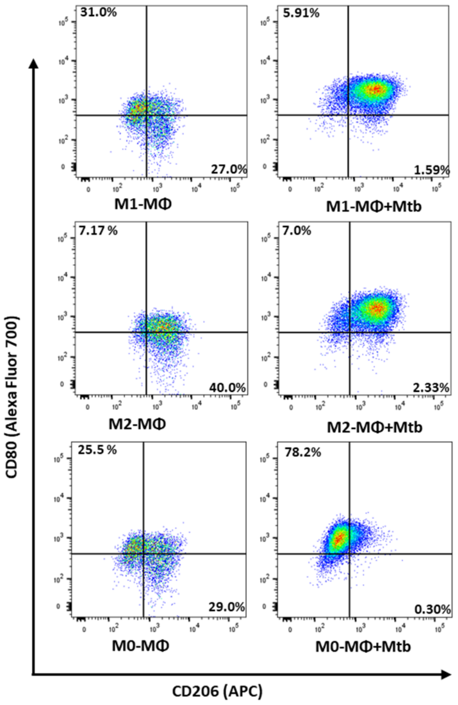

Supplemental Figure-2: Mtb infected human M1, M2 and M0-MΦs show a differential cytokine and chemokine transcript responses and protein secretion profile. a *Ex vivo* differentiated MΦs were infected with Mtb and culture supernatants collected at 18hr. post infection tested for pro and anti-inflammatory cytokines using sandwich ELISA . * < 0.01, **< 0.009, *t* test. **b** *Left panels*: RNAseq analysis of naïve or Mtb infected M1- and M2-MΦs shows a differential expression of CC and CXC chemokine genes. Fragments Per Kilobase of transcript per Million mapped reads (FPKM, mean± SD; n=2) and log2-fold differences are shown. *Right panels*: FPKM were analyzed for significance using Students two tailed test. * < 0.05. **c** Elisa quantitated Chemokines differentially expressed by M1-, M2- and M0-MΦs are shown. * < 0.01, **< 0.009, *t* test. Note enhanced IL-1β correlated with enhanced gene expression for Caspases in Mtb infected M1- MΦs vs. M2-MΦs are shown (see SF-5).

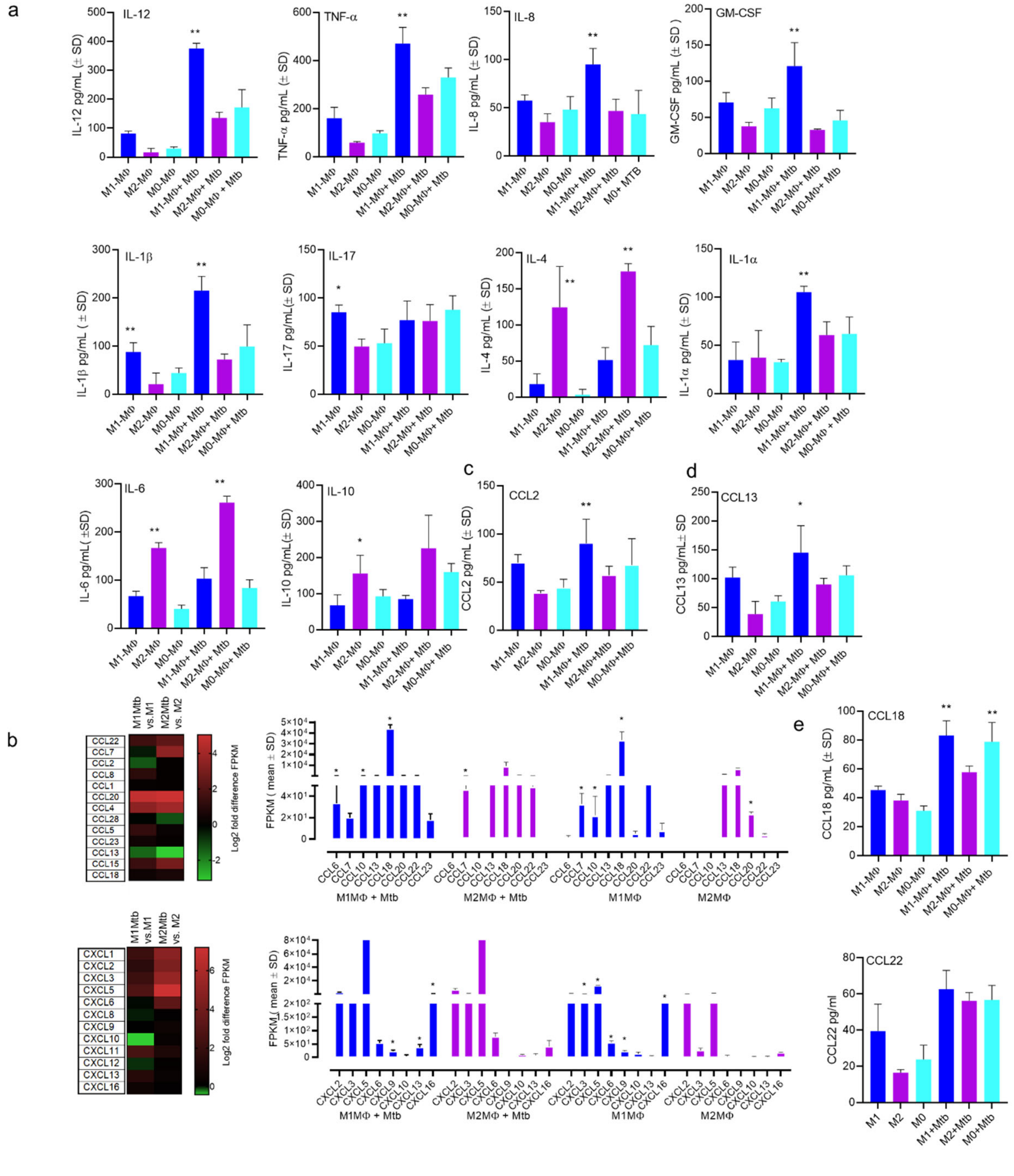

Supplemental Figure-3a: Mtb infected human M1, M2 and M0-MΦs but not naïve MΦs show an upregulation of iNOS protein. *Ex vivo* differentiated MΦs were infected with Mtb and lysates probed for iNOS using Wes-Proteinsimple blot system (Abclonal iNOS ab (A0312). Left: Blot profiles indicated; Middle: Densitometry is shown. Right: qPCR of mRNA for iNOS shown. * p < 0.01 t test.

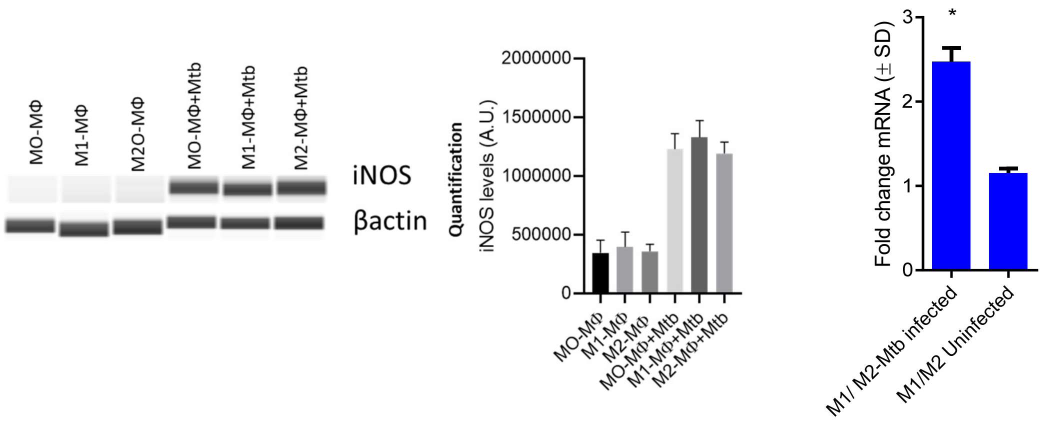

Supplemental Figure-3b: Mtb infected human M1, M2 and M0-MΦs show a differential reactive oxygen species secretion. *Ex vivo* differentiated MΦs were infected with Mtb and cells treated incubated in 1 mM dichloro-dihydro-fluorescein diacetate followed by reading RLUs using a fluorometer at 18hr. post infection. **< 0.007, *t* test. Left: whole cell ROS using DCFDA; Right: Mitochondrial ROS detected using MitoRos (*p < 0.01 using two tailed t test).

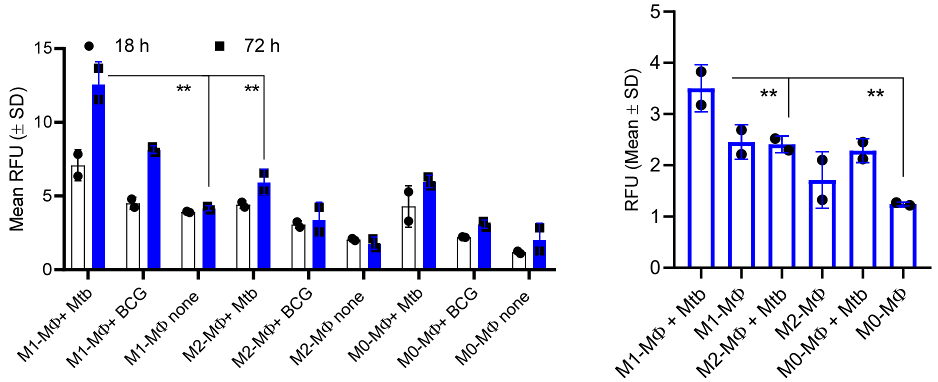

Supplemental Figure-3c: Mtb infected human M1, M2 and M0-MΦs show a differential enrichment of LAMP1 on Mtb phagosomes. Macrophages infected with gfpMtb (LAMP1) or rfpMtb (Rab7) were labeled using antibodies to LAMP1 and Rab7 followed by confocal imaging. Related to Fig.1m (LAMP1) and Fig.4b (Rab7). Quantitation of localization is shown in main Figures.

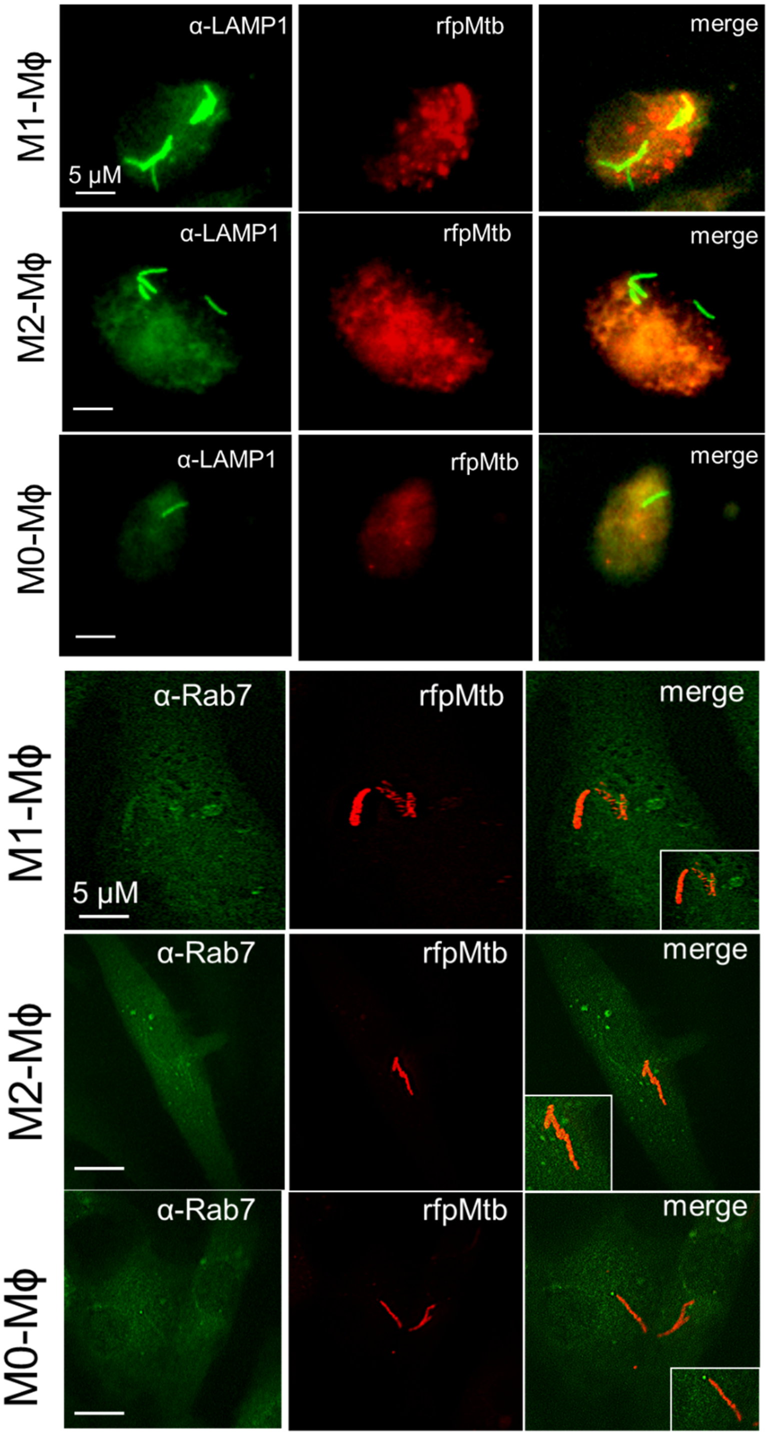

Supplemental Figure-4: Differential gene expression (DEG) analysis of Mtb infected M1-, M2- and M0-MΦs. Only selected genes of >top ~100 upregulated genes which showed > log2 change in expression are shown.

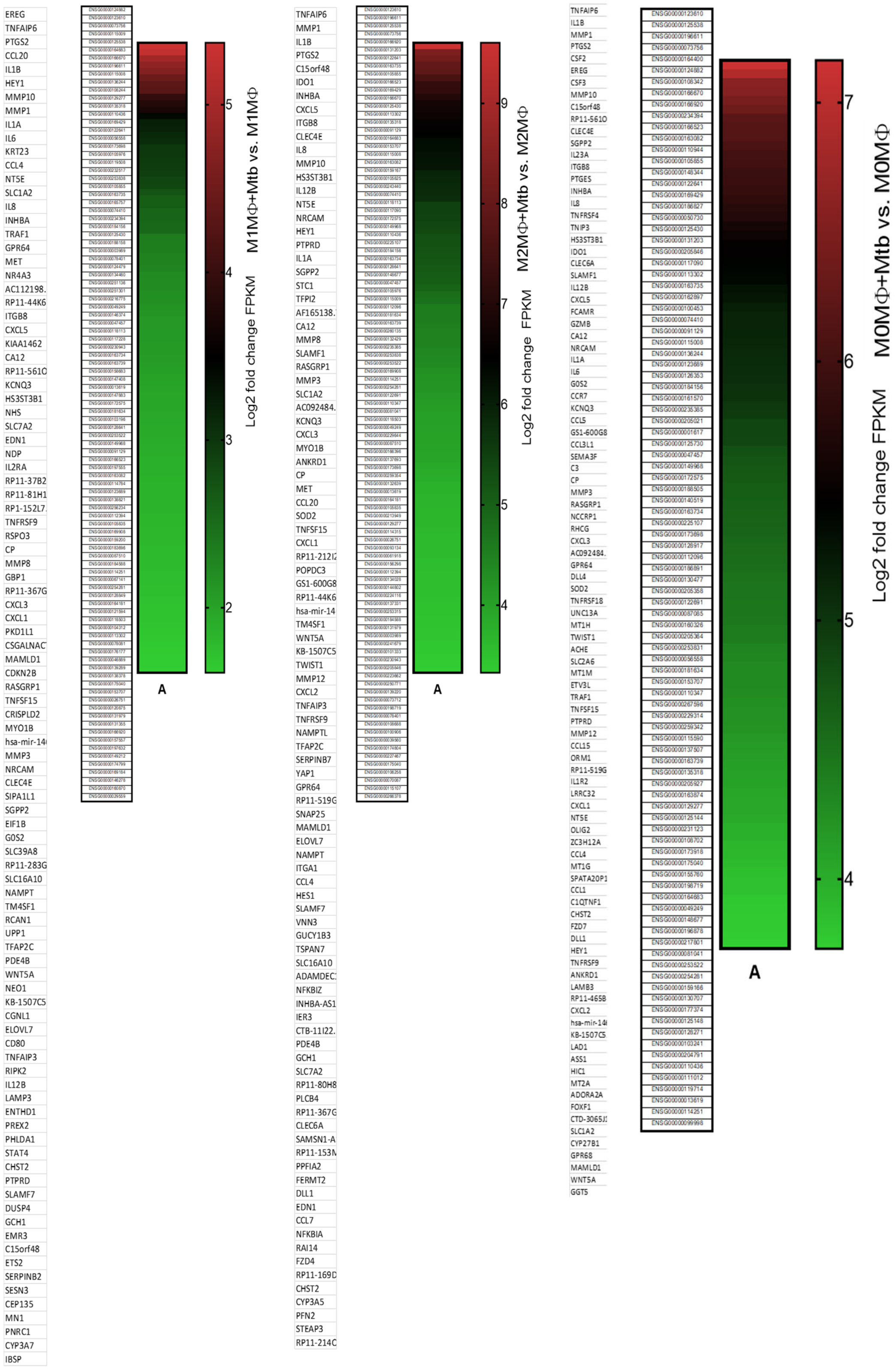

Supplemental Figure-5: **Down-regulation of expression in Mtb infected M1-, M2- and M0-MΦs.** Fig. 2 main text shows up-regulation of gene expression. Gene clusters which are 'down regulated' are shown for the same groups. (* $p < 0.0001$, *Clusterprofile* workflow. Novogene Inc, USA).

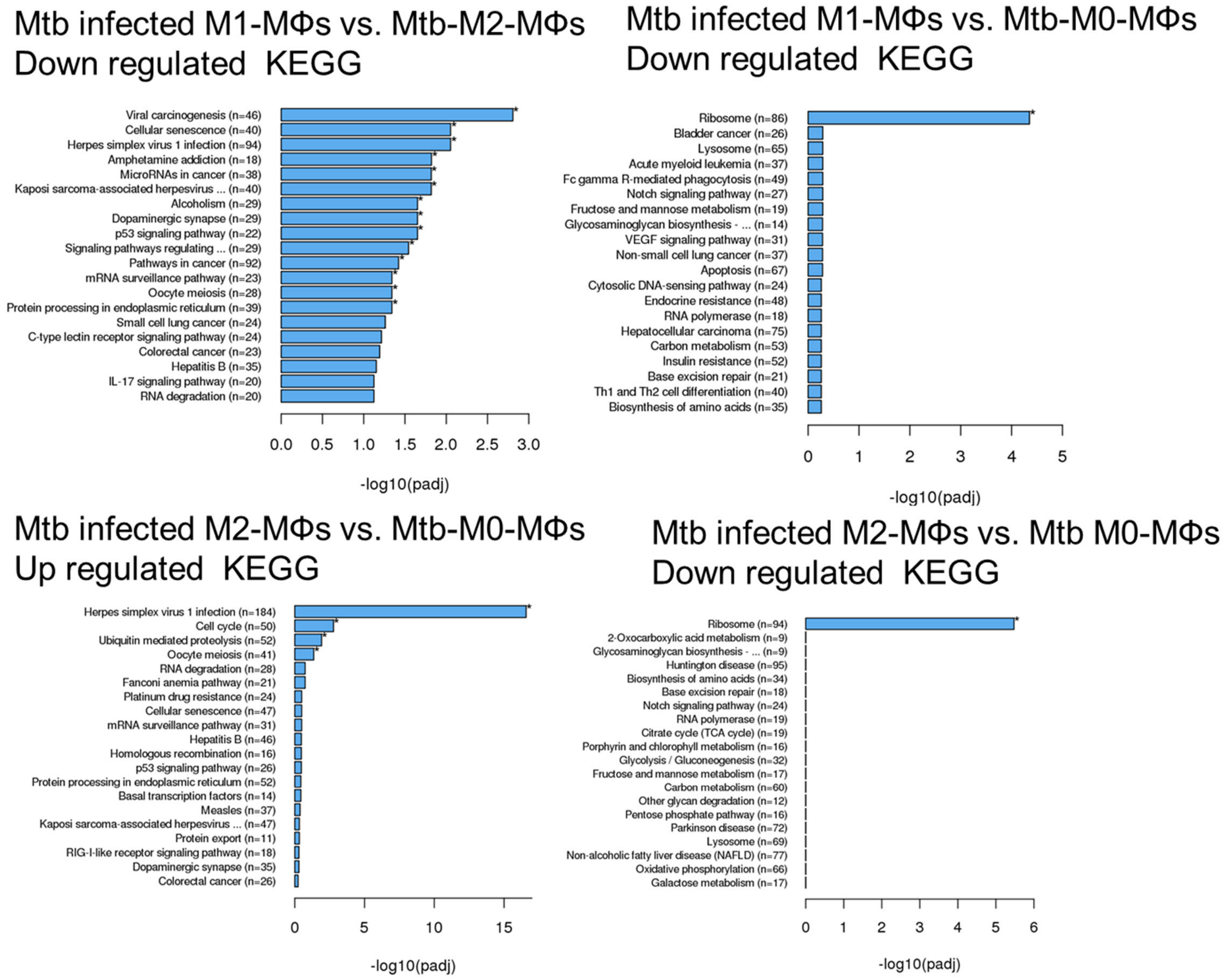

Supplemental Figure-6: *Clusterprofile* pathway analysis of gene expression in Mtb infected M1 and M2-MΦs. *NOD- receptor* mediated signaling is illustrated. *Caspase gene expression up regulated (red) in Mtb infected M1-MΦs* (highlighted box) *when enhance IL-1β secretion (SF.1).*

Mtb infected

M1-MΦs vs.

Mtb+M2-MΦs

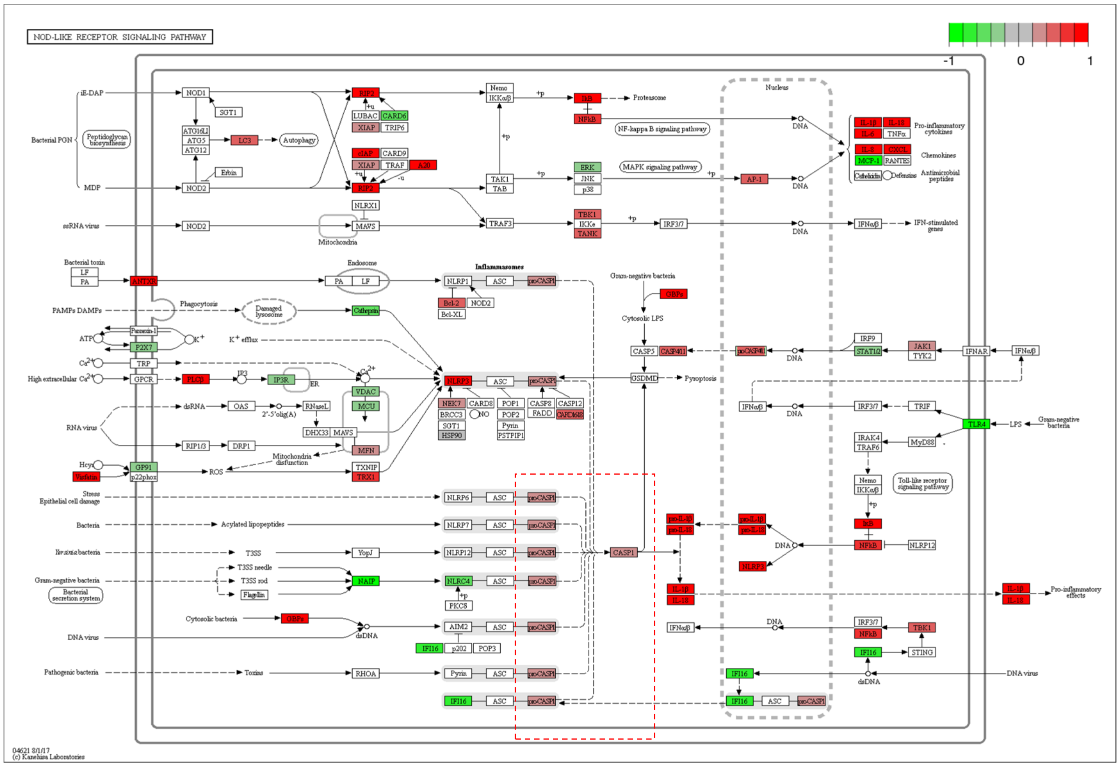

Mtb infected

M2-MΦs vs.

Mtb+M0-MΦs

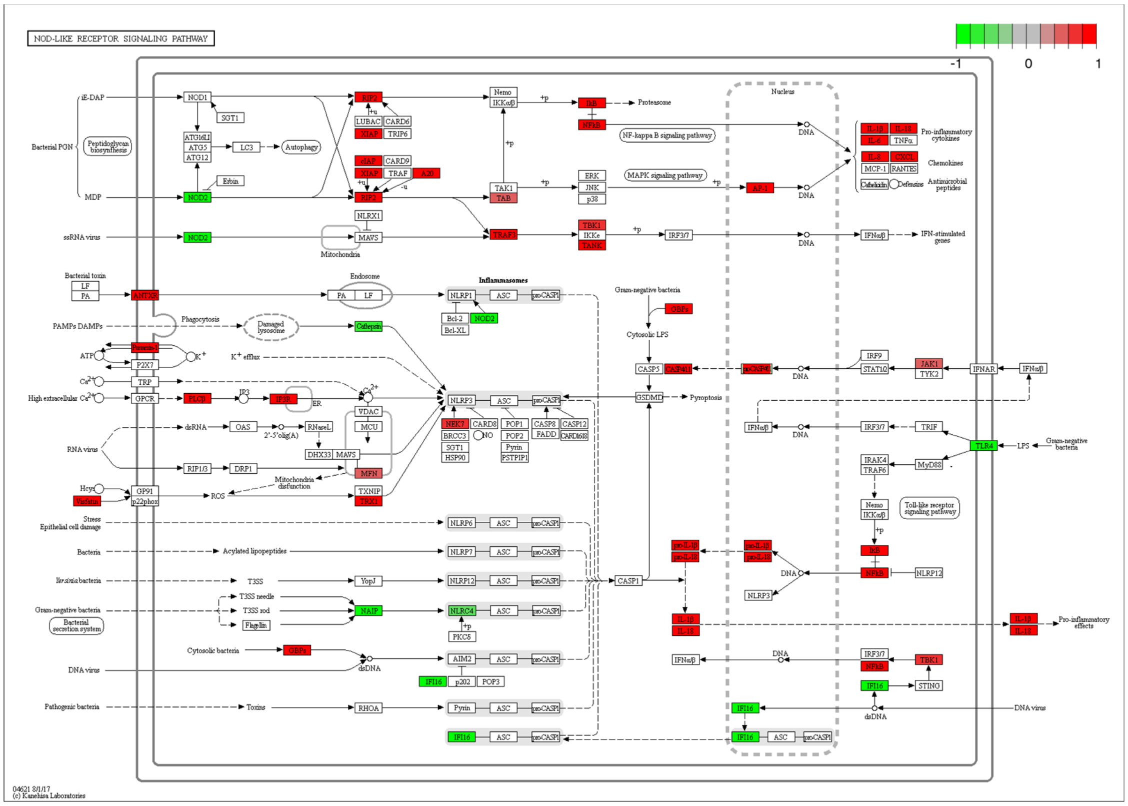

Supplemental Figure-7: *Clusterprofile* pathway analysis of 'Tuberculosis' group gene expression in Mtb infected M1, M2 and M0-MΦs.

Mtb infected M1-MΦs vs.

Mtb+M2-MΦs

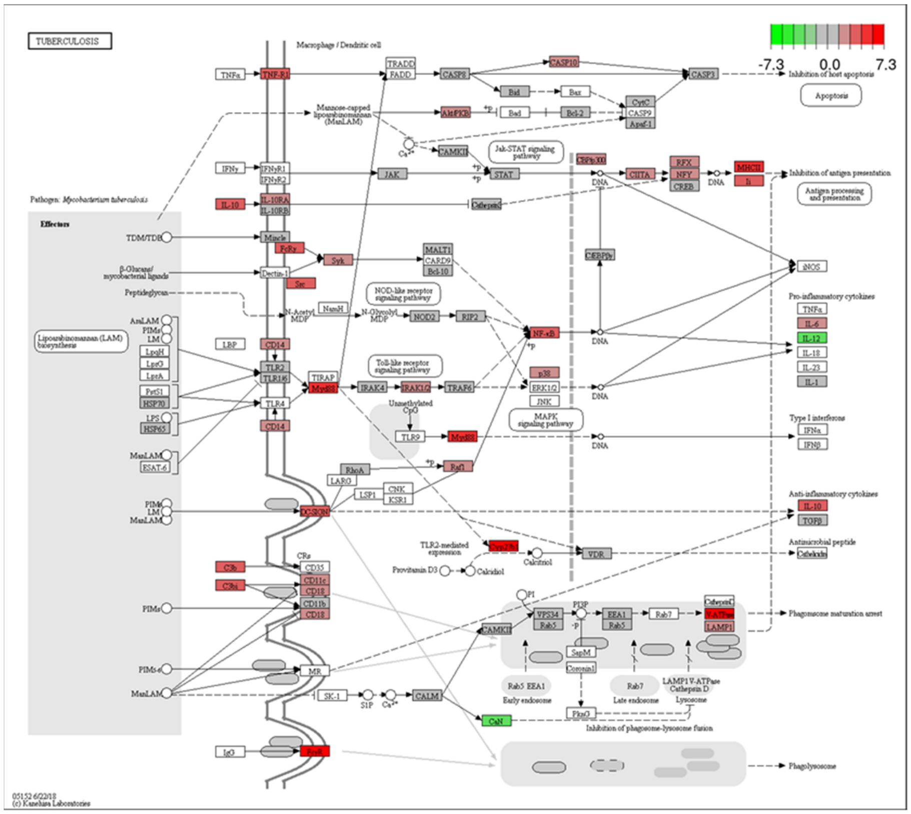

Mtb infected M2-MΦs vs.

Mtb+M0-MΦs

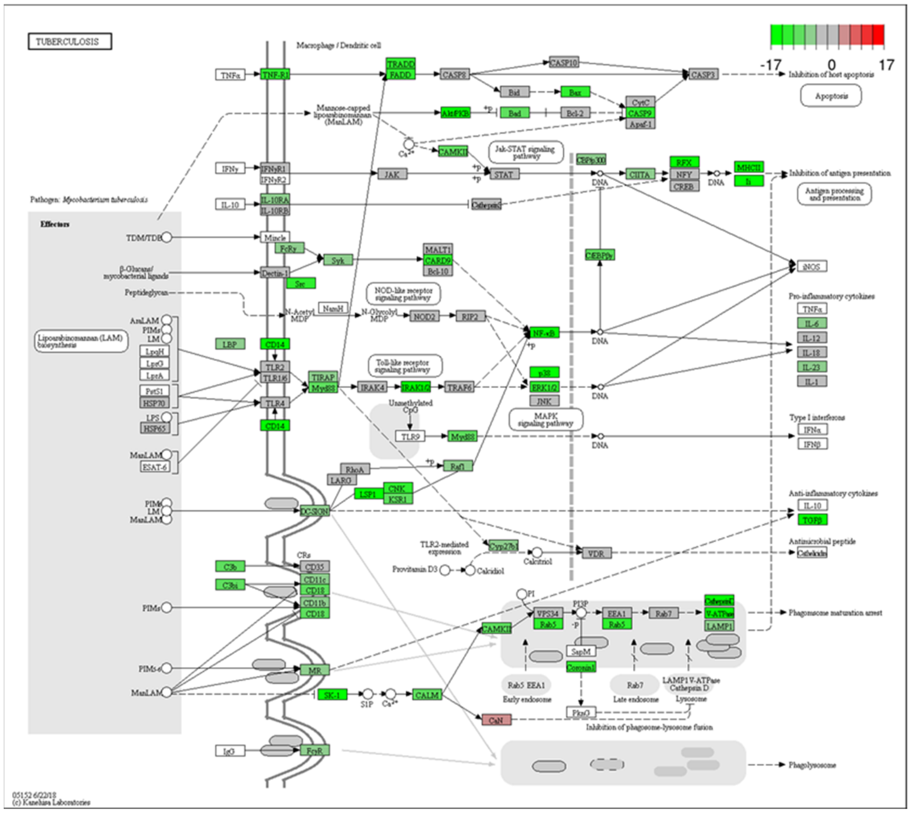

Supplemental Figure-8: '*Simple'* western blot analysis of ATG expression in Mtb infected M1, M2 and M0-MΦs (see SF-14 for '*Simple*' capillary gel electrophoresis procedure)

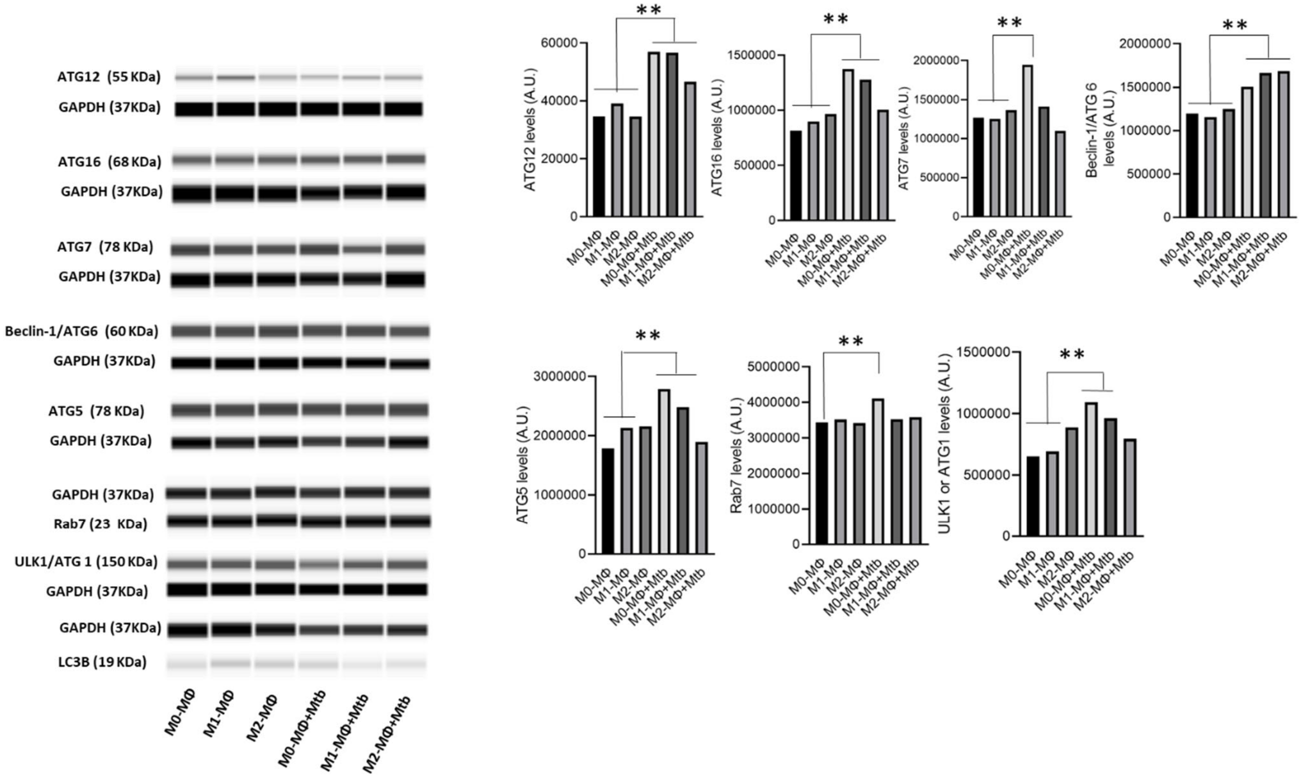

Supplemental Figure-9: *Clusterprofile* pathway analysis of antigen processing gene expression in Mtb infected M1, M2 and M0-MΦs

Mtb infected M1-MΦs vs.

Mtb+M2-MΦs

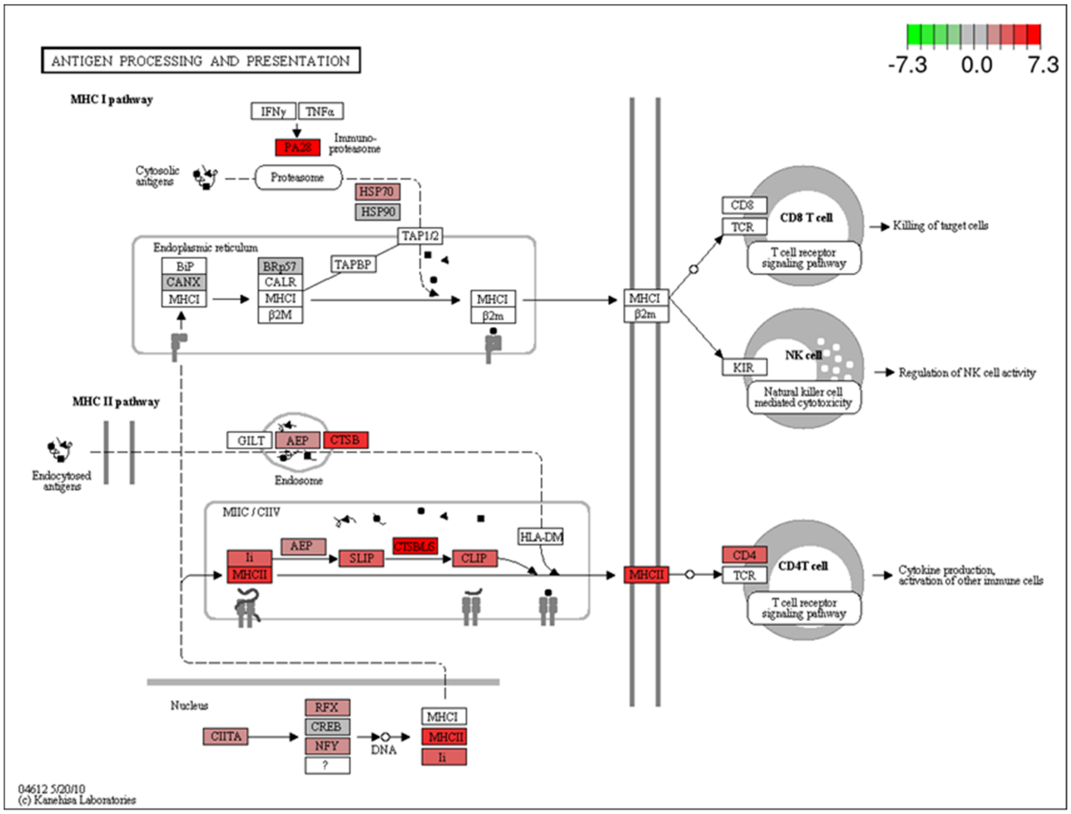

Mtb infected M2-MΦs vs.

Mtb+M0-MΦs

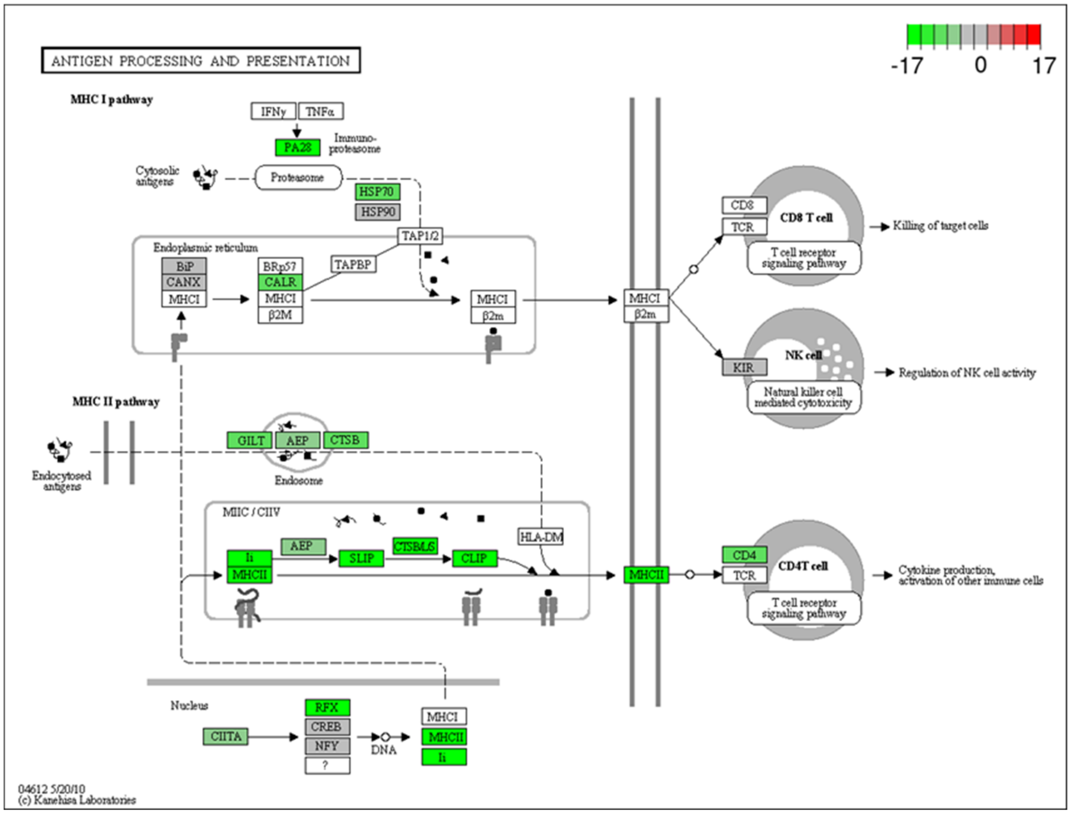

Supplemental Figure-10: *Clusterprofile* pathway analysis of Lysosomal gene expression in Mtb infected M1, M2 and M0-MΦs. Lysosomal proteases like Cathepsins, glycosidases and lipases are highlighted to the left.

Mtb infected M1-MΦs vs.

Mtb+M2-MΦs

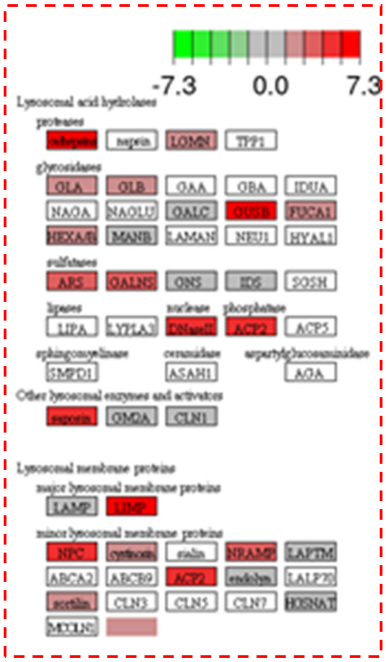

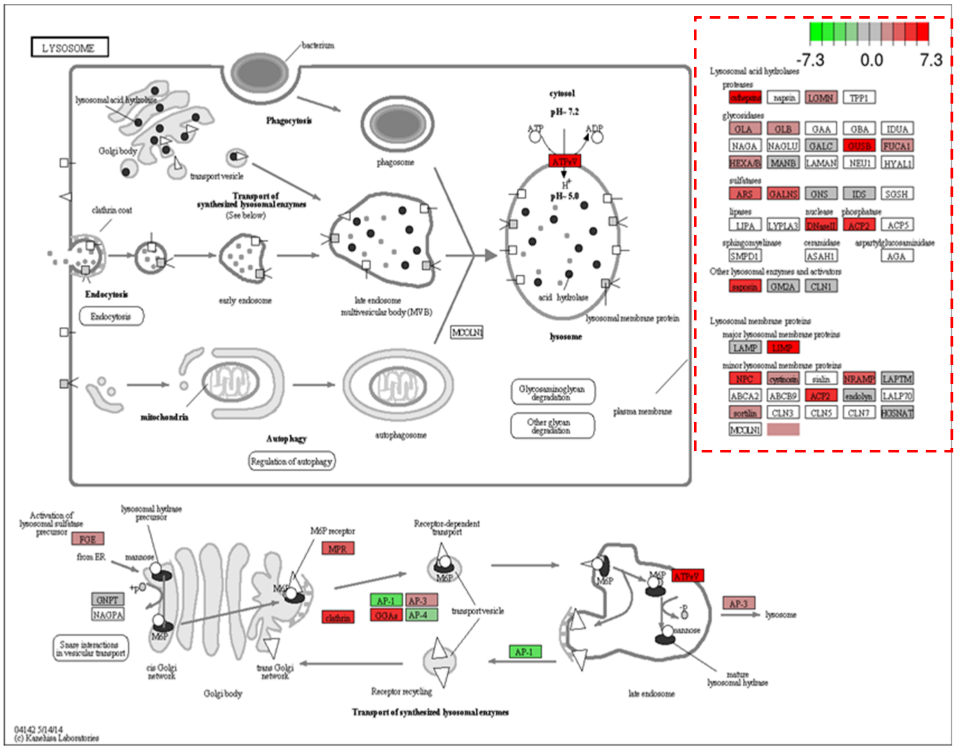

Mtb infected M2-MΦs vs.

Mtb+M0-MΦs

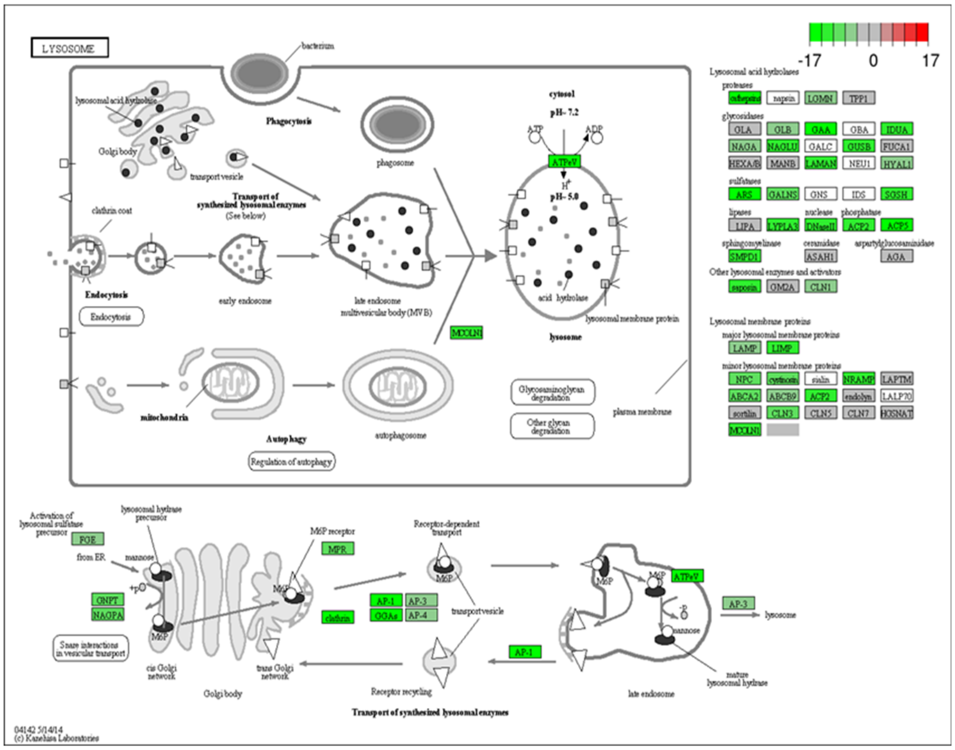

Supplemental Figure -11: Biomarker genes and TFs which are differentially expressed during the transcriptomics of human blood from TB patients and LTBI (8 studies compared; Singhania et al, 2018, *Nature Imm*, 19,1159). Distribution of these genes shown in M1, M2 and M0-MΦs before and after infection with Mtb from this study. Values are Mean FPMK (n=2) (+ indicate expression in MΦs; - indicates no expression in MΦs).

Genes /Gene cluster Observed in > 2 studies	**Mean FPKM in Mtb/naive infected M1-MΦs**	**Mean FPKM in Mtb infected/naive M2-MΦs**	**Mean FPKM in Mtb infected/naive M0-MΦs**
DUSP3	1678.818 /2202.335	953.3249 /2049.629	3621.01 /6002.957
FC -gamma receptor cluster: FCGR1A, FCGR1B, FCGR1C	Figure 6	Figure 6	Figure 6
SEPTIN cluster: SEPT4	Not detected	Not detected	67.14166 /74.24991
SEPT2 (this study only)	5345.322 /7134.336	5300.023 /9916.673	3276.358 /5748.287
SEPT11 (this study only)	6430.194 /5451.145	5257.346 /4038.622	3080.334 /2493.353
GBP cluster:GBP4 GBP5 GBP1 GBP 2 GBP6 GBP3, GBP5 (this study only)	Figure 6	Figure 6	Figure 6
ANKRD2	411.4292 /442.3031	254.5118 /288.9497	164.0981 /116.6086
BATF2	0.534486 /0	0 /1.842238	94.27638 /149.0838
GAS6	32.1491 /30.35582	10.602 /1.348186	433.1825 /1655.422
SERPING1	56.3941 /62.53262	0 /0.921119	784.9096 /741.3397
LHFPL2	11136.43 /13807.33	4210.608 /3550.984	4736.907 /3404.546
S100A8	3880.484 /7612.845	20.36729 /142.8964	10158.9 /5858.476
Genes /Gene cluster Observed in one of 8 studies			
SCARF1	21.70551 10.90318	30.49339 5.909124	4735.546 1226.791
AAK1	+	+	+
ALDH1A1	-	-	-
APOL1 ; APOL4	+	+	+++
ARG1	+	+	+
ARHGEF9	-	-	-
ARNTL	-	-	-
BACH2	-	-	-
BDH1	+	+	+
BLK 1	-	-	-
BTN3A1	-	-	-
C19ORF; C19ORF; C1QB	+	+	+

C4ORF18; C5; C1QC			
CALML4	-	-	-
CASC1	-	-	-
CCDC120	+	+	+
CCR6	+	+	+
CD177	-	-	-
CD1C	+	+	+
CD274	++	++	++
CD74	+	-	+++
CD79A ; CD79B	-	-	-
CD96	+	+	+
CERKL	+	+	+
CLC	+	+	+
CNIH4	+	+	+
COL4A4	-	-	-
MARCD3	-	-	-
TMCC1	+	+	+
SMARCD3	+	+	++
SMYD5	+	-	+
SPHK1	-	-	+
STAT1	++	++	+
TAP1	+	-	++
TMEM25	-	-	-
TRAF4	-	-	+
TRAFD1	+	-	+
TRIM47	-	-	+
UGP2	++	++	++
USP54	+	+	+
VAMP5	-	-	+
VEGFB 1	-	-	-
VPREB3	-	-	-
ZNF296	++	++	++

Supplemental Figure-12: *Clusterprofiler* analysis of a lymph node transcriptome from Mb infected neonatal macaques (One of three NHP LNs analyzed shown; similar profiles for 2 others not shown). Red indicates up-regulated; green down-regulated.

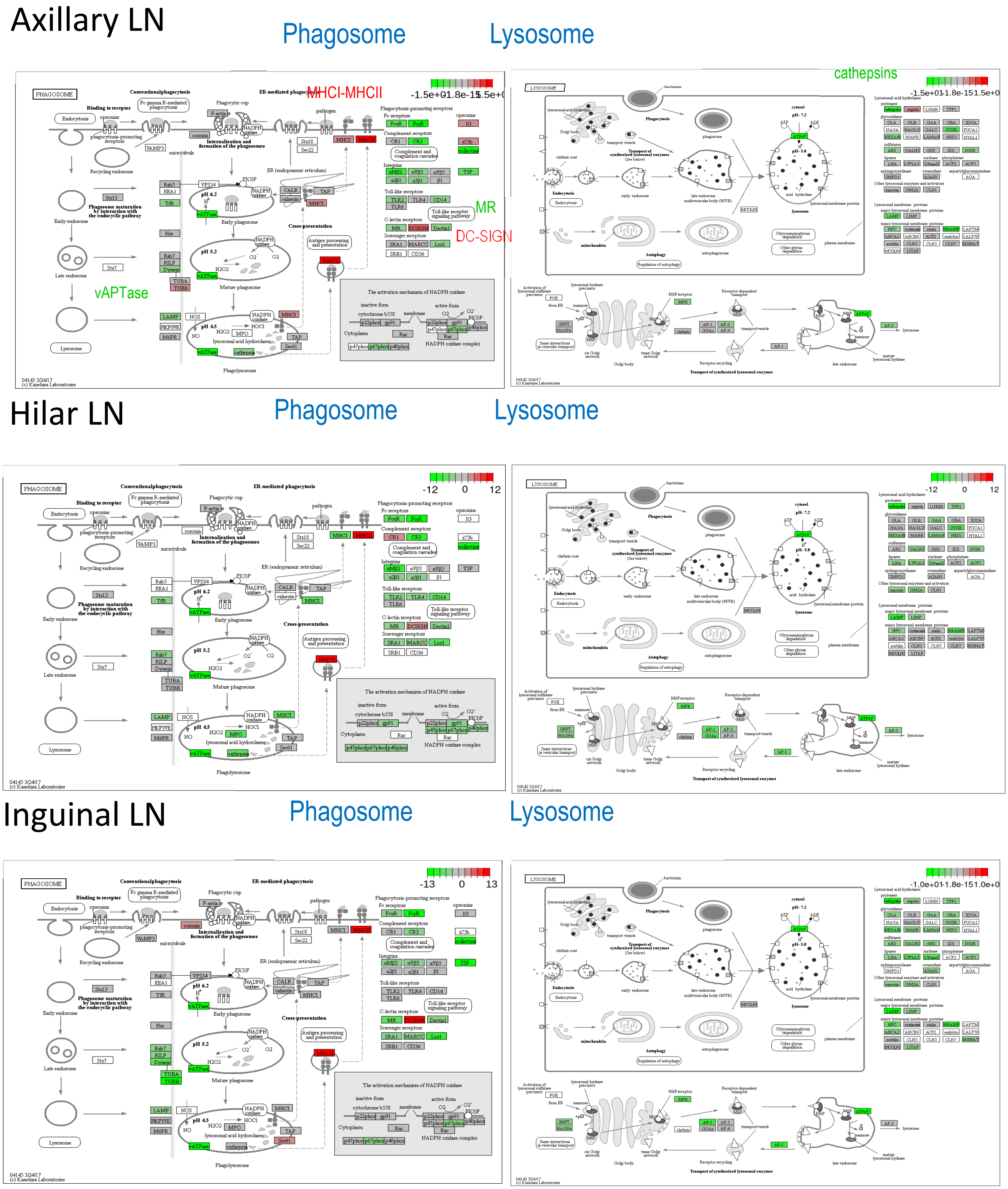

Supplemental Figure-13:

Novogene | Leading Edge Genomic Services & Solutions

THIS SECTION IS BEING PROVIDED FOR REVIEW PURPOSES ONLY: RNA-sequencing of Mtb infected Macrophages; these were conducted two times following the strategy.

readings the p values (< 01 Library Preparation and Sequencing

From the RNA sample to the final data, each step, including sample test, library preparation, and sequencing, influences the quality of the data, and data quality directly impacts the analysis results. To guarantee the reliability of the data, quality control (QC) is performed at each step of the procedure. The workflow is as follows:

Total RNA qualification

mRNA enrichment

Double-stranded cDNA synthesis

End repair, poly-A&adaptor addition

Fragments selection and PCR

Library quality assessment

Illumina sequencing

1.1 Total RNA Sample QC

All samples need to pass through the following three steps before library construction:

(I) Nanodrop: preliminary quantitation

(2) Agarose Gel Electrophoresis: tests RNA degradation and potential contamination

(3) Agilent 2100: checks RNA integrity and quantitation

Novogene | Leading Edge Genomic Services & Solutions

1.2 Library Construction and Quality Assessment

After the QC procedures, mRNA from eukaryotic organisms is enriched from total

1

RNA using oligo(dT) beads. For prokaryotic samples, rRNA is removed using a specialized kit that leaves the mRNA. The mRNA from either eukaryotic or prokaryotic sources then fragmented randomly in fragmentation buffer, followed by cDNA synthesis using random hexamers and reverse transcriptase. After first-strand synthesis, a custom second-strand synthesis buffer (Illumina) is added, with dNTPs, RNase H and Escherichia coli polymerase I to generate the second strand by nick-translation and AMPure XP beads is used to purify the cDNA. The final cDNA library is ready after a round of purification, terminal repair, Atailing, ligation of sequencing adapters, size selection and PCR enrichment. Library concentration was first quantified using a Qubit 2.0 fluorometer (Life Technologies), and then diluted to I ng/gl before checking insert size on an Agilent 2100 and quantifying to greater accuracy by quantitative PCR (Q-PCR) (library activity >2 nM). The workflow chart is as follows:

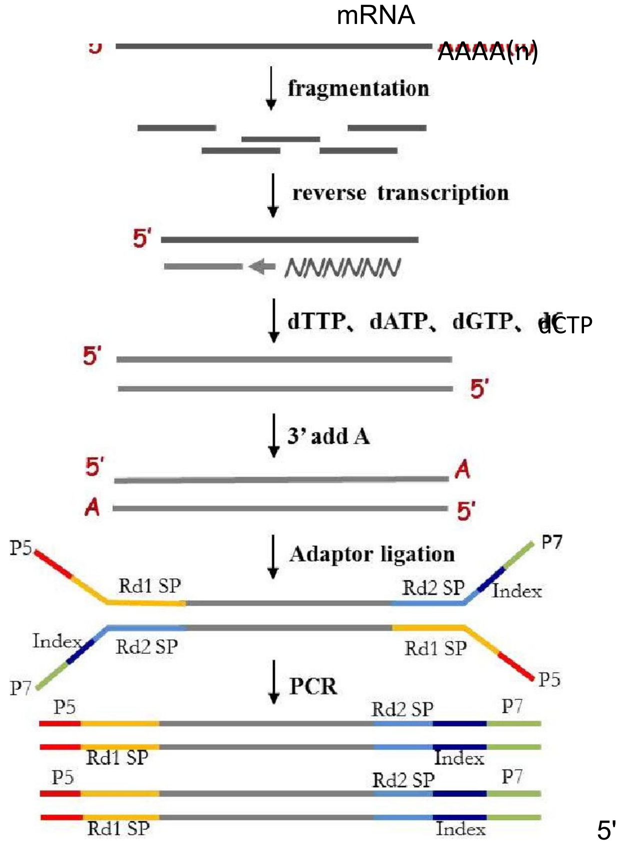

1.3 Sequencing

Libraries are fed into Novaseq6000 machines according to activity and expected data volume. A paired-end 150 bp sequencing strategy was used and all samples were sequenced to at least 6 Gb.

Supplemental Table 1

SiRNAs (Duplexes Custom synthesized by Origene)

Target	Source	Catalog No.	Dilution
Atg5	OriGene	SR322789	1/1000
Beclin 1/Atg6	OriGene	SR322490	1/1000
Atg7	OriGene	SR323157	1/1000
Atg12	OriGene	SR306046	1/1000
Atg16L1	OriGene	SR324356	1/1000
ULK1/Atg1	OriGene	SR322391	1/1000
IFITM3	OriGene	SR323108	1/1000
SLAMF8	OriGene	SR324597	1/1000
GABRAP	OriGene	SR307771	1/1000
AMBRA1	OriGene	SR310808	1/1000
Rab7	GE Dharmacon	NC1660768	1/1000

Antibodies used for Flow Cytometry

Target	Source	Catalog No.	Dilution
PE-Cy7 anti-human CD68	BD Biosciences	565595	1/1000
PE anti-human CD14	Invitrogen	12-0149-42	1/1000
APC anti-human CD206	Biolegend	321110	1/1000
AF700 anti-human CD80	BD Biosciences	561133	1/1000
Aqua fluorescent reactive dye	Invitrogen, cat no	L34957	1/1000

Antibodies used for Western blot

Target	Source	Catalog No.	Dilution
IFITM3	Cell Signaling	59212S	1/50
ATG12	Cell Signaling	2010P	1/50
ATg16L	Cell Signaling	8089s	1/50
ATG7	Cell Signaling	8558s	1/50
Beclin-1	Cell Signaling	3738s	1/50
ATG5	Cell Signaling	12994s	1/50
Rab7	Cell Signaling	9367T	1/50
ULK1	Cell Signaling	8054s	1/50
LC3B	Cell Signaling	2775s	1/300
Gbp1	Novus Biologicals	NBP-1-31560	1/50
Sirtuin Antibody Sampler Kit	Cell Signaling	9787T	1/50
Class I HDAC Antibody Sampler Kit	Cell Signaling	65816T	1/50
Class II HDAC Antibody Sampler Kit	Cell Signaling	79891T	1/50
SQSTM1 Polyclonal Antibody	abclonal	A19700	1/50
Rab8	Cell Signaling	6975T	1/50
NDP52	Cell Signaling	60732S	1/50
GAPDH	Sigma	SAB5600208	1/50

GBP5	Cell Signaling	67798S	1/50
FCgRA	abclonal	A2552	
Standardized for MILO protein analysis in single cells (Fig. 3 main text)			
ATG7:	R&D	MAB6608	1/10
ATG5:	Cell signaling	12994s	1/10
GAPDH:	Cell Signaling	5174S	1/10
N-Acetyl-L-leucyl-L-leucyl-L-methional (NLALLM); Cathepsin pan in inhibitor	Tocris biotechne	0384/10	
Balicatib; Cathepsin K inhibitor	Tocris biotechne	5585/10	
E 64; Cysteine protease inhibitor	Tocris biotechne	5208/10	
Calpeptin; Cathepsin-L (CTSL) inhibitor	Tocris biotechne	0448/10	
Sirtinol: sirtuin inhibitor	Tocris biotechne	3521/10	
Tubastatin A hydrochloride HDAC inhibitor	Tocris biotechne	6270/10	

Supplemental Table 2. Sequences of ATG Primers for Q-RT-PCR

Primer Set	*Sequence (5'- 3')* **Forward**	*Sequence (5'- 3')* **Reverse**
ATG1	ATGGGTCTAGGCGACCATCT	GCTGCTGTTGATTTGCACCA
ATG3	TCCCATGTGTTCAGTTCACCC	TGCCACTAATCTTACATACAGGG
ATG4A	TGCTGGTTGGGGATGTATGC	GCGTTGGTATTCTTTGGGTTGT
ATG4B	ATGGACGCAGCTACTCTGAC	TTTTCTACCCAGTATCCAAACGG
ATG5	AAAGATGTGCTTCGAGATGTGT	CACTTTGTCAGTTACCAACGTCA
ATG6 (Beclin1)	GGTGTCTCTCGCAGATTCATC	TCAGTCTTCGGCTGAGGTTCT
ATG7	CAGTTTGCCCCTTTTAGTAGTGC	CCAGCCGATACTCGTTCAGC
ATG8E (LC3)	AACATGAGCGAGTTGGTCAAG	CGTGTTCGCTCTACTGCCC
ATG12	CTGCTGGCGACACCAAGAAA	GATGATGAATGCGAGTCAGATGC
ATG13	TTGCTATAACTAGGGTGACACCA	CCCAACACGAACTGTCTGGA
ATG14	GCGCCAAATGCGTTCAGAG	AGTCGGCTTAACCTTTCCTTCT
ATG16L1	AACGCTGTGCAGTTCAGTCC	AGCTGCTAAGAGGTAAGATCCA
SQSTM1 (p62)	GCACCCCAATGTGATCTGC	CGCTACACAAGTCGTAGTCTGG
hPPIA	CCCACCGTGTTCTTCGACATT	GGACCCGTATGCTTTAGGATGA

qPCR assay for gene expression in human and macaque macrophages

Total RNA was extracted using RNAeasy mini kit (Qiagen, Germany) from human and NHP PBMCs or M1/M2 polarized macrophages. RNA concentration and purity ratios (OD260/280, OD260/230) were measured using the NanoDrop ND-1000 spectrophotometer (Thermo Fisher

Scientific, USA). cDNA synthesis was performed on a CFX96 Real-Time PCR System (Biorad, USA) using the 2X OneStep qRT-PCR Mastermix Kit (Applied Biosystems, USA) according to manufacturer's instruction. Quantitative PCR (qPCR) was performed using SYBR green probe and gene specific primers (Table 1 for Human and Table2 for NHP). Threshold cycle numbers were transformed to $\Delta\Delta C_t$ values, and the results were expressed relative to the reference gene, β-actin and GAPDH. Gene expression data was performed using GraphPad Prism ver. 6.0 suite (GraphPad Software). Student's t-test was used for means comparison between both uninfected and Mtb infected M1- and M2-polarised cells. Significance was set at the 0.05 level.

Supplemental Table 3. qPCR primers for Human

GENE	Forward primer 5'->3'	Reverse primer 5'->3'
**GBP 1**	GTGGAACGTGTGAAAGCTGA	CAACTGGACCCTGTCGTTCT
**GBP 2**	GATTTCACCCTGGAACTGGA	GGGTTCAGCTCTTCCTCCTT
**GBP 3**	TTAATCTGCCCCGACTCTGT	CATTGACCTTGATGCCTCCT
**GBP 5**	CAGGAACAACAGATGCAGGA	TCATCGTTATTAACAGTCCTCTGG
**GBP 6**	TCCTGTGCAGCACCTTTGTC	AGCTCCGTCACATAATGCAGC
**Siglec 14**	GCACCTGTCCTTCATCCTTTC	AGGAGCCCTGCTGTTTCTCA
**Siglec 15**	CGCGGATCGTCAACATCTC	GTTCGGCGGTCACTAGGTG
**SlamF1**	AGGCCCTCCACGTTATCTA	GCAAAAGCGCTGAACTGA
**SlamF7**	TCTCTTTGTACTGGGGCTATTTC	TTTTCCATCTTTTTCGGTATTT
**SlamF9**	TGGTGTGCTCTGTGGAGAAG	TTTTCTGGACTCGGATGACC
**iNOS**	CAGCGGGATGACTTTCCAAG	AGGCAAGATTTGGACCTGCA

Supplemental Table 4. qPCR primers for NHP

Gene	Forward primer 5'->3'	Reverse primer 5'->3'
**Siglec14**	GCACCTGTCCTTCATCCTTTC	AGGAGCCCTGCTGTTTCTCA
**GBP 2**	TCAATCATATGCGACTCCATTTTG	AGGGAAACCTGGGATGAGATTTAG
**SIRT2**	CAGAACATAGATACCCTGGAGCGA A	AAGGTCCTCCAGCTCCTTCTTC
**SIRT5**	CGAGTCGTGGTCATCACCCAGAAC ATC	ACTCTTGTAATTCTCAGCCACAACTCC AC
**SLAMF1**	AGGCCCTCCACGTTATCTA	GCAAAAGCGCTGAACTGA
**HDAC1**	GTCCAGATAACATGTCGGAGTACA GC	CGATGTCCGTCTGCTGCTTATTAAG
**HDAC2**	CCTCATAGAATCCGCATGACCCATA AC	AGACATGTTATCTGGTCTTATTGACCG TAG
**cathepsin B**	TTCTTGCGACTCTTGGGACTTC	TGACGAGGATGACAGGGAACTA
**cathepsin D**	CCCACACACACCCACACACTCG	CCAGGGAGGGGAAAACCACAGA
**cathepsin L**	AGGGTCAGTGTGGTTCTTGTTG	TGAGATAAGCCTCCCAGTTTTC

Fold change is calculated by using $2^{-\Delta\Delta Ct}$ method; Briefly:

ΔCt = Ct (gene of interest) – Ct (housekeeping gene)

ΔΔCt = ΔCt (treated sample) – ΔCt (untreated sample)

Fold change = $2^{-\Delta\Delta Ct}$

Supplemental Figure-14: 'Protein Simple' Western Assay / Wes analysis of proteins in lysates of Mtb infected vs naïve macrophages. For the analysis of protein levels of macrophages, we used the quantitative Wes capillary immunoassay, in which proteins were separated and detected using Wes separation capillary cartridge 12-230 kDa along with Wes Anti-Rabbit Detection Module (Simple Western system and Compass Software, Protein Simple). In brief, glass microcapillaries were loaded with stacking and separation matrices followed by sample loading. During capillary electrophoresis, proteins were separated by size and then immobilized to the capillary wall. Samples were loaded at 1 mg/ml dilution and the primary rabbit antibodies and GAPDH were used at 1:50 dilution. Data were analyzed with the Compass software (version 2.6.7). The area under the curve (AUC), which represents the signal intensity of the chemiluminescent reaction was analyzed for all the antibodies and GAPDH. Values given for protein expression were normalized to GAPDH. Quantitation of protein levels (area under each peak; arbitrary units [A.U.]) were performed using the Compass software (version 2.6.7). All the primary antibodies used for the Simple Western were as follows: IFITM3 (cat. no.59212S), ATG12 (cat. no. 2010P), ATg16L (cat. no. 8089s), ATG7 (cat. no. 8558s), Beclin-1 (cat. no. 3738s), ATG5 (cat. no. 12994s), Rab7 (cat. no. 9367T), ULK1 (cat. no. 8054s), LC3B (cat. no. 2775s), GAPDH (cat. no. 5174s) and Gbp1 (cat. no. NBP-1-31560). All the antibodies were purchased form cell signaling except for Gbp1 which was purchased from Novus biologicals. *Nature communications* https://doi.org/10.1038/s41467-018-08247-x.ttps://doi.org/10.1038/s41467-018-08247-x

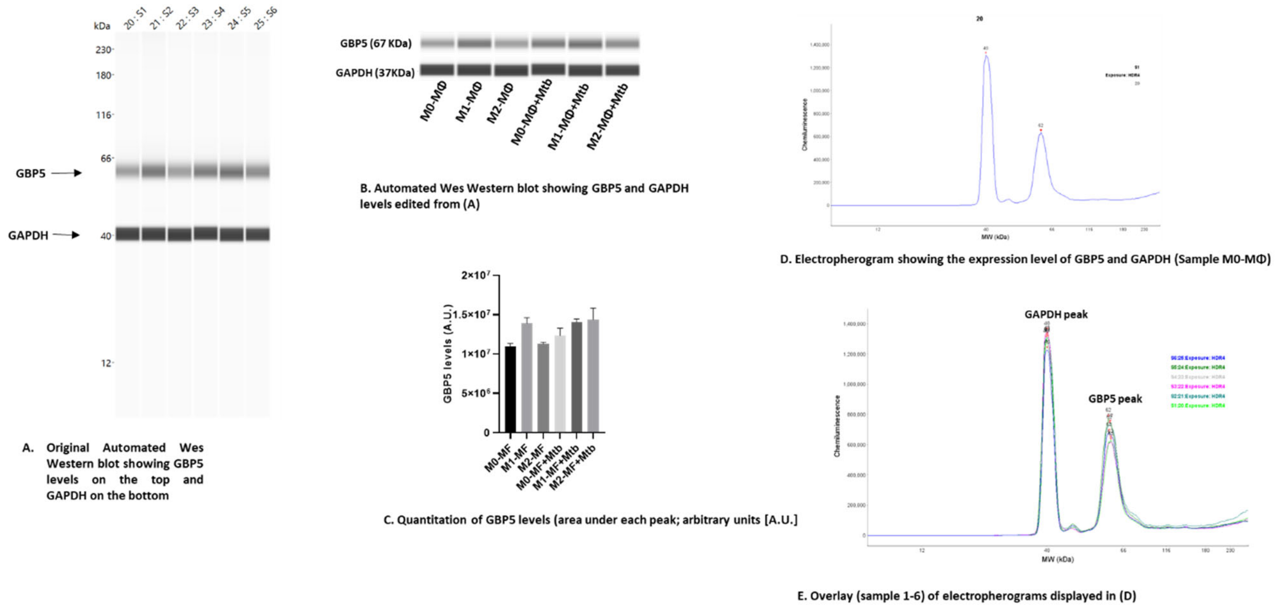

A. Original Automated Wes Western blot showing GBP5 levels on the top and GAPDH on the bottom

B. Automated Wes Western blot showing GBP5 and GAPDH levels edited from (A)

C. Quantitation of GBP5 levels (area under each peak; arbitrary units [A.U.]

D. Electropherogram showing the expression level of GBP5 and GAPDH (Sample M0-MΦ)

E. Overlay (sample 1-6) of electropherograms displayed in (D)

Supplemental Figure-15: Densitometry of blots:

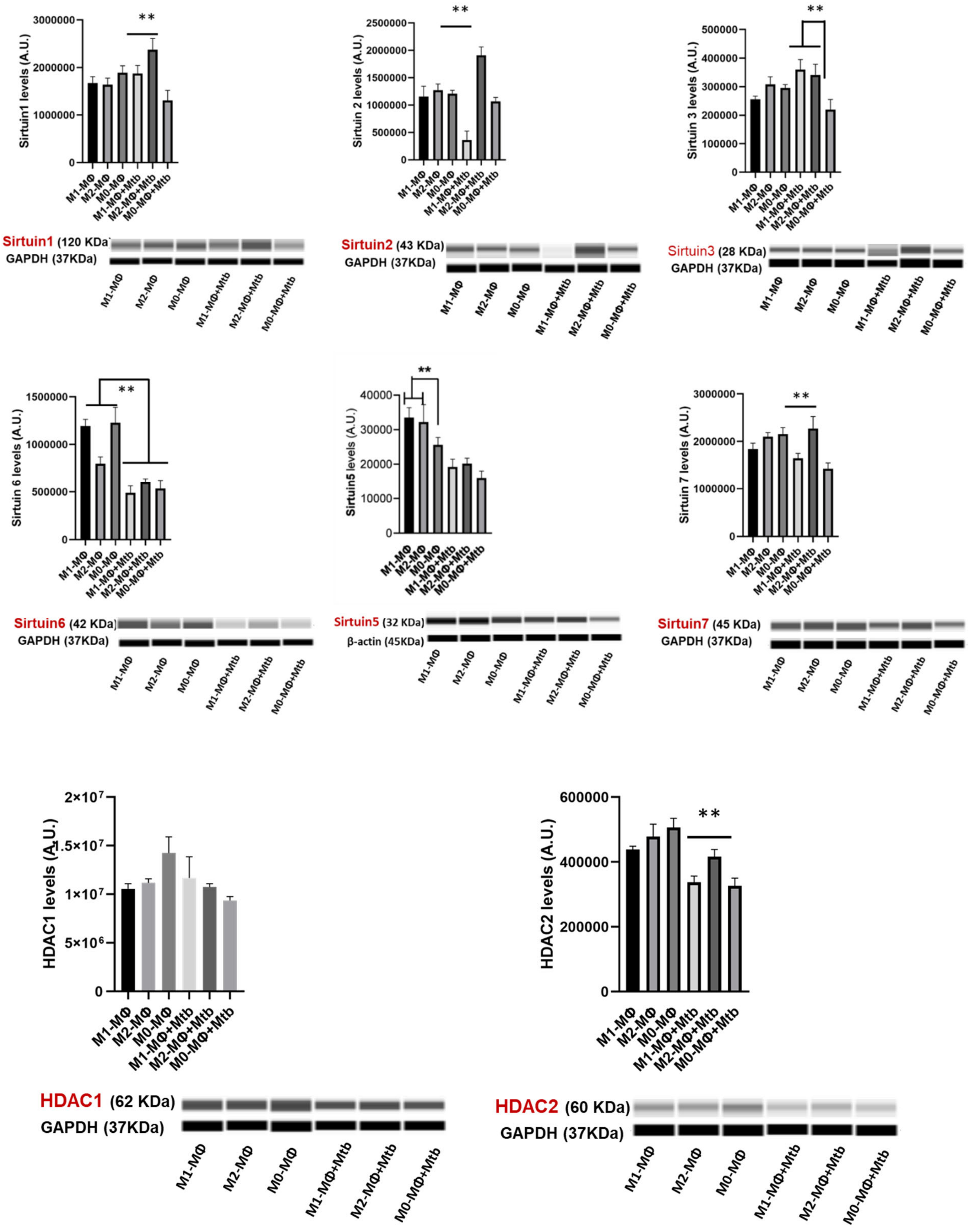

Densitometry of blots Fig. 6: Western (Wes) Blot analysis of Proteins associated with Antigen Processing. Densitometry readings with the p values (< 0.01) for comparisons within groups (naïve vs. Mtb infected). Proteins for which reliable antibodies were available are shown. Fujiwara et al show GBP5 is enriched in M1-MΦs which is reproduced here although after Mtb infection, GBP5 levels are uniformly increased (see also Fig. 4 main text). SQSTM1 is an autophagy substrate which gets depleted during autophagic flux. FcRs are ideally detected using flow cytometry on the surface of macrophages although protein levels of cell lysates may differ.

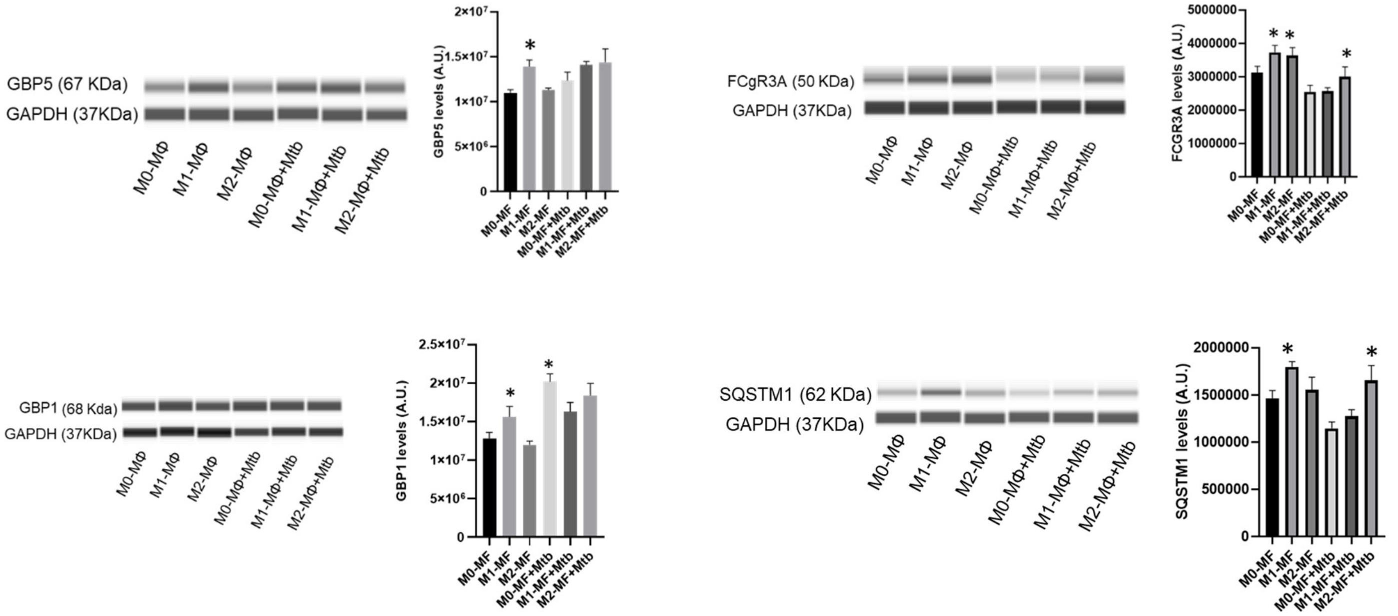

Supplement: Supplementary file 1 — Supplementary Information [file 42003_2022_3387_MOESM1_ESM.pdf]
