# Supplementary material for: Human M1 macrophages express unique innate immune response genes after mycobacterial infection to defend against tuberculosis
Source: Commun Biol. 2022 May 19;5:480. doi: 10.1038/s42003-022-03387-9 (PMC9119986; doi:10.1038/s42003-022-03387-9)
Supplement: Supplementary file 2 — Description of Additional Supplementary Files [file 42003_2022_3387_MOESM2_ESM.pdf]

# Description of Additional Supplementary Files

**File name:** Supplementary Data 1
**Description:** Source data table.
