# Supplementary material for: Human M1 macrophages express unique innate immune response genes after mycobacterial infection to defend against tuberculosis
Source: Commun Biol. 2022 May 19;5:480. doi: 10.1038/s42003-022-03387-9 (PMC9119986; doi:10.1038/s42003-022-03387-9)
Supplement: Supplementary file 4 — Reporting Summary [file 42003_2022_3387_MOESM4_ESM.pdf]

nature portfolio

Corresponding author(s): CHINNASWAMY JAGANANTH.PH.D.

Last updated by author(s): Dec 30, 2021

Reporting Summary

Nature Portfolio wishes to improve the reproducibility of the work that we publish. This form provides structure for consistency and transparency in reporting. For further information on Nature Portfolio policies, see our Editorial Policies and the Editorial Policy Checklist.

Statistics

For all statistical analyses, confirm that the following items are present in the figure legend, table legend, main text, or Methods section.

n/a	Confirmed	
☐	☒	The exact sample size (n) for each experimental group/condition, given as a discrete number and unit of measurement
☐	☒	A statement on whether measurements were taken from distinct samples or whether the same sample was measured repeatedly
☐	☒	The statistical test(s) used AND whether they are one- or two-sided<br>*Only common tests should be described solely by name; describe more complex techniques in the Methods section.*
☒	☐	A description of all covariates tested
☒	☐	A description of any assumptions or corrections, such as tests of normality and adjustment for multiple comparisons
☒	☐	A full description of the statistical parameters including central tendency (e.g. means) or other basic estimates (e.g. regression coefficient) AND variation (e.g. standard deviation) or associated estimates of uncertainty (e.g. confidence intervals)
☒	☐	For null hypothesis testing, the test statistic (e.g. F, t, r) with confidence intervals, effect sizes, degrees of freedom and P value noted<br>*Give P values as exact values whenever suitable.*
☒	☐	For Bayesian analysis, information on the choice of priors and Markov chain Monte Carlo settings
☒	☐	For hierarchical and complex designs, identification of the appropriate level for tests and full reporting of outcomes
☒	☐	Estimates of effect sizes (e.g. Cohen's d, Pearson's r), indicating how they were calculated

Our web collection on statistics for biologists contains articles on many of the points above.

Software and code

Policy information about availability of computer code

Data collection	NA
Data analysis	NA

For manuscripts utilizing custom algorithms or software that are central to the research but not yet described in published literature, software must be made available to editors and reviewers. We strongly encourage code deposition in a community repository (e.g. GitHub). See the Nature Portfolio guidelines for submitting code & software for further information.

Data

Policy information about availability of data

All manuscripts must include a data availability statement. This statement should provide the following information, where applicable:

- Accession codes, unique identifiers, or web links for publicly available datasets
- A description of any restrictions on data availability
- For clinical datasets or third party data, please ensure that the statement adheres to our policy

Data availability statement has been included in the main text.

Field-specific reporting

Please select the one below that is the best fit for your research. If you are not sure, read the appropriate sections before making your selection.

☒ Life sciences ☐ Behavioural & social sciences ☐ Ecological, evolutionary & environmental sciences

For a reference copy of the document with all sections, see nature.com/documents/nr-reporting-summary-flat.pdf

Life sciences study design

All studies must disclose on these points even when the disclosure is negative.

Sample size	Graphpad prism was used to calculate significance and the required n per experiment.
Data exclusions	Data sets were not excluded.
Replication	All experiments contained triplicate or duplicate biological replicates and were repeated 2-3 at separate time points.
Randomization	Most studies used donor derived macrophages which were randomly collected from healthy donors. 10 sampes were collected form PBMCs of children with TB and without TB from a field site.
Blinding	Most experiments were coded to perform experiments blind; performing tech or PDFs were not aware of the effect of drugs or identity of samples. PBMCs from children were coded for a blind tPCR esting by a PDF.

Reporting for specific materials, systems and methods

We require information from authors about some types of materials, experimental systems and methods used in many studies. Here, indicate whether each material, system or method listed is relevant to your study. If you are not sure if a list item applies to your research, read the appropriate section before selecting a response.

Materials & experimental systems

n/a	Involved in the study	
☐	☒	Antibodies
☒	☐	Eukaryotic cell lines
☒	☐	Palaeontology and archaeology
☐	☒	Animals and other organisms
☐	☒	Human research participants
☒	☐	Clinical data
☒	☐	Dual use research of concern

Methods

n/a	Involved in the study	
☒	☐	ChIP-seq
☐	☒	Flow cytometry
☒	☐	MRI-based neuroimaging

Antibodies

Antibodies used	All antibodies used have been listed in supplemental information and are validated in other publications.
Validation	All antibodies were are validated in other publications listed at the source of the supplier.

Animals and other organisms

Policy information about studies involving animals; ARRIVE guidelines recommended for reporting animal research

Laboratory animals	NHP Models were used (rhesis monkeys at age 6 weeks.
Wild animals	na
Field-collected samples	na
Ethics oversight	NHP Models were used under an approved IACUC protocol from Texas biomedical center, (# 1655-MM, Texas Biomedical Center) San antonio Texas USA

Note that full information on the approval of the study protocol must also be provided in the manuscript.

Human research participants

Policy information about studies involving human research participants

Population characteristics	Healthy identified donors derived blood samples or TB patients studiesdunder ongoing protocols were analyzed. Ten samples of children with or whtotu tB were collected under approved protocols.
Recruitment	They were a part of ongoing studies.
Ethics oversight	Samples were collected under protocols:PBMC collected from children #527/QD-BVPTU of the Director of the National Lung Hospital (NLH), NLH Institutional Review Board (IRB), Vietnam; PBMCs from US-border zone under HSC-SPH-12-0037, UT School of public health, Brownsville, TX, USA.

Note that full information on the approval of the study protocol must also be provided in the manuscript.

Flow Cytometry

Plots

Confirm that:

☒ The axis labels state the marker and fluorochrome used (e.g. CD4-FITC).

☒ The axis scales are clearly visible. Include numbers along axes only for bottom left plot of group (a 'group' is an analysis of identical markers).

☒ All plots are contour plots with outliers or pseudocolor plots.

☒ A numerical value for number of cells or percentage (with statistics) is provided.

Methodology

Sample preparation	Described under methods
Instrument	Fortessa flow cytometer (Beckton Dickinson),
Software	FlowJo v10 software (Tree Star, Inc.).
Cell population abundance	Described under methods
Gating strategy	Described under supplementary infomation

☒ Tick this box to confirm that a figure exemplifying the gating strategy is provided in the Supplementary Information.
